# Effects of Ferric Oxyhydroxide on Anaerobic Microbial Dechlorination of Polychlorinated Biphenyls in Hudson and Grasse River Sediment Microcosms: Dechlorination Extent, Preferences, *Ortho* Removal, and Its Enhancement

**DOI:** 10.3389/fmicb.2018.01574

**Published:** 2018-07-20

**Authors:** Yan Xu, Kelvin B. Gregory, Jeanne M. VanBriesen

**Affiliations:** ^1^Department of Municipal Engineering, School of Civil Engineering, Southeast University, Nanjing, China; ^2^Department of Civil and Environmental Engineering, Carnegie Mellon University, Pittsburgh, PA, United States

**Keywords:** polychlorinated biphenyls, sediment, reductive dechlorination, electron acceptor, ferric oxyhydroxide, *Dehalococcoides*

## Abstract

Microbial reductive dechlorination of polychlorinated biphenyls (PCBs) has been observed in many PCB-impacted sediments. However, this biodegradation is relatively site-specific and can be affected by PCB compositions and sediment geochemical conditions. To better understand the influence of a common competing electron acceptor, ferric oxyhydroxide (FeOOH), on dechlorination, two sediments (Hudson River and Grasse River sediments), and two PCB mixtures (PCB 5/12, 64/71, 105/114, and 149/153/170 in *Mixture 1* and PCB 5/12, 64/71, 82/97/99, 144/170 in *Mixture 2*) were used for this microcosm study. The addition of 40 mmole/kg FeOOH completely inhibited PCB dechlorination in the Hudson sediment, but only moderately inhibited PCB dechlorination in the Grasse sediment with a 3-week longer lag time. The inhibitory effect in the Grasse sediment was mainly due to the loss of unflanked *para* dechlorination activity. Fe(II) analysis showed that dechlorination started prior to the consumption of Fe(III), which indicates PCB reduction and Fe(III) reduction were able to take place concurrently. *Dehalococcoides* 16S rRNA genes increased with the commencement of dechlorination in the Grasse sediment, but not in the completely inhibited Hudson sediment. Rare *ortho* dechlorination pathways were identified in FeOOH-amended Grasse sediment microcosms, dominated by transformations of PCB 25(24-3-CB) to PCB 13(3-4-CB) and PCB 28(24-4-CB) to PCB 15(4-4-CB). The addition of carbon sources (acetate or a fatty acid mixture with acetate, propionate, and butyrate) after 27 weeks of incubation reinitiated dechlorination in FeOOH-amended Hudson sediment microcosms. Also, the addition of carbon sources greatly enhanced *ortho* dechlorination in FeOOH-amended Grasse microcosms, indicating the utilization of acetate and/or the fatty acid mixture for *ortho* dechlorination-related microorganisms. A dechlorination pathway analysis approach revealed that *para*-flanked *meta* dechlorination was primarily preferred followed by *ortho-*/double-flanked *meta* dechlorination and single-/double-flanked *para* dechlorination in the Grasse sediment.

## Introduction

Polychlorinated biphenyls (PCBs), containing 209 individual congeners, are listed as persistent organic pollutants (POPs) by the Stockholm Convention (UNEP, 2001). They have 1–10 chlorine atoms substituted at 4 *ortho*, 4 *meta*, and 2 *para* sites on the biphenyl structure. Although the industrial production of PCBs was banned in late 1970s, the global occurrence of PCBs in the environment has raised concerns about potential negative effects on aquatic systems and human health (Brown et al., 1987; Safe, 1989; Quensen et al., 1998; IARC Monographs, 2016; Lyall et al., 2017). Hudson and Grasse Rivers are two historically PCB-impacted rivers in the United States. They received discharges from the capacitor manufacturing plants of the General Electric Company (GE) and an aluminum smelting and fabricating facility of the Aluminum Company of America (ˆAlcoa), respectively (EPA, 1997). Because PCBs are highly hydrophobic and persistent, they are predominantly adsorbed in sediment upon release into the aquatic environment and then enter the food chain via ingestion by benthic organisms (Kjellerup et al., 2008). As sediments are the sinks for PCBs in the environment, the remediation of PCB-contaminated sediments has been a regulatory priority for over three decades (EPA, 1997; Sowers and May, 2013).

Among the remediation methods, microbially mediated anaerobic reductive dechlorination of PCBs is particularly attractive due to its low cost and minimal disruption of the sediment (Bedard and Quensen, 1995; Sowers and May, 2013). Despite the prevalence of PCB dechlorination reported in a variety of natural sediment systems and laboratory microcosms, lack of knowledge of the interactions among sediment geochemical properties and dechlorinating microbial communities has hindered the progress of *in situ* treatment (Brown et al., 1987; Alder et al., 1993; Wiegel and Wu, 2000; Kjellerup et al., 2008; Xu et al., 2012; Sowers and May, 2013; Praveckova et al., 2016). In general, different sediments proceed to dechlorination following eight classified processes (Bedard and Quensen, 1995; Bedard et al., 2005; Hughes et al., 2010). However, the link among sediment properties, processes and dechlorinating microorganisms is not well understood. To date, only a few PCB dechlorinators within the *Chloroflexi* phylum have been identified. Bacterium *ortho*-17 (*o*-17), isolated from Baltimore Harbor sediment, is the only strain that is able to remove *ortho* chlorines (Cutter et al., 1998, 2001). *Dehalobium chlorocoercia* strain DF-1 growing with a *Desulfovibrio* sp. as co-species catalyzes PCBs with double-flanked chlorines (Wu et al., 2002), *Dehalococcoides mccartyi* strains 195, CBDB1, CG-1, CG-4, CG-5, and JNA were all found to preferentially target flanked (double-flanked or single-/double-flanked) *meta*/*para* chlorines (Fennell et al., 2004; Adrian et al., 2009; Wang and He, 2013; Laroe et al., 2014; Wang et al., 2014; Zhen et al., 2014). None of the identified PCB dechlorinators shows a dechlorination preference on unflanked chlorines. Additionally, among all these PCB dechlorinators, only *D. mccartyi* strains CBDB1 and JNA were found to match known dechlorination processes H and N, respectively (Adrian et al., 2009; Laroe et al., 2014). Therefore, the observed preferences (patterns) in most PCB dechlorination studies have not been attributed to the identified dechlorinators. Moreover, sediment geochemical conditions have been found to exhibit a significant impact on dechlorination preferences (Cho and Oh, 2005; Yan et al., 2006; Kjellerup et al., 2008). Among the geochemical properties, iron is crucial for sediment biogeochemistry. Fe (III) oxides can serve as alternative electron acceptors and inhibit reductive dechlorination (Morris et al., 1992; Paul et al., 2013), while surface-complexed Fe(II) can induce abiotic dechlorination (Jeong et al., 2011; Chen et al., 2016). Abiotic PCB dechlorination associated with biogenic absorbed Fe(II) was expected in PCB-spiked acetate-amended sediment reactors applied with low-voltage electric fields (Liu et al., 2017). Up to now, little is known about the impact of Fe(III) on PCB dechlorination extent and dechlorination pathway preferences in sediments. Organic carbon is also of great importance in PCB dechlorination. A previous review concluded that the impacts of exogenous organic carbon sources, such as acetate, formate, pyruvate, lactate, glucose, methanol, acetone, and a fatty acid mixture (acetate, propionate, and butyrate) on PCB dechlorination rate, extent, and process were equivocal (Wiegel and Wu, 2000). The addition of carbon sources can benefit PCB dechlorination by providing sufficient carbon and energy sources for PCB dechlorinating microorganisms, or by stimulating the growth of essential co-species of PCB dechlorinators (Wiegel and Wu, 2000). Yet, supplementary carbon can also result in the rapid growth of non-PCB dechlorinating microorganisms, which may ultimately inhibit PCB dechlorinators through competition (Wiegel and Wu, 2000; Zanaroli et al., 2012a). General conclusions are difficult to draw due to the variety of experimental conditions considered. In some cases, researchers add the carbon sources at the initiation of incubation (Pulliam Holoman et al., 1998; Fagervold et al., 2011; Ho and Liu, 2012; Kaya et al., 2017), while others add carbon periodically during incubation (Alder et al., 1993; Zhen et al., 2014; Matturro et al., 2016). No study has considered long-term incubation of PCB spiked sediment microcosms prior to carbon addition. Accordingly, the role of exogenous carbon sources when natural carbon may have been depleted (over long times) has not been evaluated. Also, carbon sources are expected to compensate the “electron donor demand” exhibited by amended Fe(III) (Wei and Finneran, 2011). In our previous study, by developing a data analysis approach focusing on chlorine neighboring conditions, we interpreted different PCB dechlorination extent and preferences in relatively low organic carbon content (1.26%) and low Fe (5310 mg/kg dry wt.) Hudson sediment compared with high organic carbon content (5.73%) and high Fe (18,000 mg/kg dry wt.) Grasse sediment. We reported on rare *ortho* dechlorination in the Grasse sediment (Xu et al., 2016). These lead to a hypothesis that Fe and carbon content may be the important factors for PCB dechlorination in these sediments.

In the present work, we investigate the effects of exogenous ferric oxyhydroxide (FeOOH) with a concentration relevant to background total Fe on PCB dechlorination in the two sediment systems, again with spiked PCB mixtures that allow us to focus on dechlorination preferences. The specific objectives were to (1) understand changes in dechlorination extent and preferences as well as changes in the populations of putative dechlorinating microorganisms; (2) validate the chlorine per biphenyl (CPB) data analysis approach focusing on chlorine neighboring conditions; (3) further investigate the occurrence of *ortho* dechlorination under Fe(III)-reducing conditions. The findings of this study improve understanding of dechlorination inhibition caused by Fe(III). Moreover, this is the first attempt to enhance rare *ortho* dechlorination using simple carbon source amendments in sediment microcosms.

## Materials and Methods

### Sediment Collection, Storage, and Characterization

Surficial sediments (the top 4 inches of sediments) were collected using a petite ponar dredge sampler from the Hudson River (N: 43°14′55.5506″; W: -073°35′37.4080″, Moreau, NY, United States) in October 2008, and the Grasse River (N:44°57′35.9577″; W: -074°48′59.8695″, Massena, NY, United States) in June 2009 as described elsewhere. The sediments were kept in filled 3-gallon plastic buckets in the dark at 4°C until used. The geochemical properties, including background PCBs, metals (e.g., Fe, Al), acid volatile sulfide, total phosphorous, total residue as percent solids, inorganic anions, and organic carbon content of the two sediments were determined previously (Xu et al., 2016).

### Experimental Set-up

Thirteen PCB congeners were used in this study based on criteria previously described (Karcher et al., 2004; Xu et al., 2016). These 13 individual PCB congeners, related to 10 tracker pairs, were classified into two mixtures. PCB *Mixture 1* was composed of congeners PCB 5(23-CB)/12(34-CB), 64(236-4-CB)/71(26-34), 105(234-34-CB)/114(2345-4-CB), and 149(236-245-CB)/153(245-245-CB)/170(2345-234-CB). PCB *Mixture 2* contained congeners PCB 5/12, 64/71, 82(234-23-CB)/97(245-23-CB)/99(245-24-CB), 144(2346-25-CB)/170. The structures of selected PCB congeners, their theoretical first step dechlorination pathways and the possibly associated dechlorination processes were discussed previously (Xu et al., 2016).

The PCB-spiked dry sediment substrates containing 500 mg/kg of each PCB mixture were prepared as described previously (Xu et al., 2016). Modified reducing anaerobic media (RAMM) using 2 mM L-cysteine⋅HCl as the reducing agent was prepared and 1% (vol/vol) of Wolfe’s vitamin solution was added (Shelton and Tiedje, 1984; Yan et al., 2006). The medium was adjusted to pH 7.0 and autoclaved for 20 min at 121°C under a nitrogen atmosphere. FeOOH stock solution (1.0 M) was synthesized following the method developed by Lovley and Phillips (1986).

Microcosms were established in an anaerobic glove box with a gas atmosphere of N_2_-H_2_ (99.1:0.9). Unless stated otherwise, 3 g PCB-spiked dry sediment substrate or dry sediment, 3 g fresh sediment inocula (dry weight basis), with or without 1.2 mmole FeOOH, and freshly prepared RAMM medium were added to a 50 ml serum bottle to achieve a total weight of 30 g. Four Fe(III)-amended PCB spiked experiments, four PCB-spiked experiments, two no PCB control experiments and sterile controls were set up (**Table 1**). Generally, a total PCB concentration of 50 mg/kg slurry was achieved in each PCB-spiked microcosm and an additional 40 mmole/kg slurry of FeOOH was added in each Fe(III)-amended PCB-spiked microcosm. Individual PCB concentrations of PCB *Mixture 1* and PCB *Mixture 2* in the sediment microcosms are tabulated in Supplementary Table S1. PCB *Mixture 1* had a CPB value of 4.36, with *ortho* CPB 1.56, *meta* CPB 1.58, and *para* CPB 1.23, while PCB *Mixture 2* had a CPB value of 4.27 with *ortho* CPB 1.64, *meta* CPB 1.48, and *para* CPB 1.16. The established microcosms were sealed with Teflon-lined gray butyl rubber septa (The West Pharmaceutical Co., PA) and crimped with aluminum crimp caps. After a 24-h rotation on a rotating mixer (40 rpm) in the dark, the first time point sampling was conducted and the rest of microcosms were stored statically at ambient temperature in the dark. Destructive sampling was conducted at 3-week intervals from Week 0 to Week 24, then at Week 30, Week 36, and Week 51 anaerobically. Unless stated otherwise, triplicate microcosms were sampled at each sampling point. The influences of carbon sources were investigated by adding acetate (pH = 7.0, sodium salt) or a fatty acid mixture (acetate/propionate/butyrate 1:1:1, pH = 7.0, sodium salts) after 27 weeks of incubation. The microcosms used for carbon source amendment were those that had been sampled and recapped at Week 21 and Week 24. At Week 27, 7.5 mM of acetate or a fatty acid mixture were added; at Week 30 and Week 36, 15 mM of acetate or a fatty acid mixture were added, respectively. To minimize the change of slurry volume, all the additions were conducted by injecting 200 times concentrated stock carbon source solutions. The sample collections for the triplicate acetate-amended microcosms and the triplicate fatty acid mixture-amended microcosm were non-destructive and performed at Week 30 and Week 36, right before the addition of extra carbon sources. The final sampling was conducted at Week 51. Sterile controls were sampled in duplicate at Week 0, Week 18, Week 36, and Week 51, and no dechlorination was observed.

**Table 1:** Sediment microcosms setup.

Experiments	Name	Sediment	Spiked PCBs	Ferric Iron
Fe(III)-amended PCB experiments	H-1-Fe	Hudson	PCB *Mixture 1*	FeOOH
	G-1-Fe	Grasse	PCB *Mixture 1*	FeOOH
	H-2-Fe	Hudson	PCB *Mixture 2*	FeOOH
	G-2-Fe	Grasse	PCB *Mixture 2*	FeOOH
				
PCB experiments	H-1	Hudson	PCB *Mixture 1*	–
	G-1	Grasse	PCB *Mixture 1*	–
	H-2	Hudson	PCB *Mixture 2*	–
	G-2	Grasse	PCB *Mixture 2*	–
				
No PCB controls	H	Hudson	–	–
	G	Grasse	–	–
				
Sterile controls (Killed controls)	H-1-Fe-K	Hudson	PCB *Mixture 1*	FeOOH
	G-1-Fe-K	Grasse	PCB *Mixture 1*	FeOOH
	H-2-Fe-K	Hudson	PCB *Mixture 2*	FeOOH
	G-2-Fe-K	Grasse	PCB *Mixture 2*	FeOOH

*PCB, polychlorinated biphenyl.*

### Headspace Analysis

Prior to microcosm slurry withdrawal, 200 μl of headspace gas was analyzed to determine H_2_, CH_4_, and trace O_2_ following an analytical method mentioned elsewhere (Xu et al., 2016). Considering biological gas production, the amount of each gas species was recalculated with the ideal gas law by simply assuming a constant 1.0 atm partial pressure of nitrogen in the headspace and a room temperature of 20°C.

### Ferrous Iron Analysis

The reduction of Fe(III) to Fe(II) in the microcosms was tracked using a ferrozine method described elsewhere (Sorensen, 1982). Briefly, a subsample of 200 μl of sediment slurry was weighed and transferred into a 1.5-ml microcentrifuge tube containing 800 μl of 1.0 M HCl and reacted thoroughly. Thereafter, 10 μl of acid treated sample was added to 4.99 ml ferrozine solution and filtered. Dilutions with more ferrozine solution were performed when necessary. The absorption was measured at 562 nm by a UV-vis spectrophotometer (HACH, Macon, MO, United States).

### PCB Extraction, Cleanup, and Analysis

The PCB extraction procedure was described elsewhere (Yan et al., 2006; Xu et al., 2016). Briefly, 2.0 g sediment slurry was collected anaerobically, and 1 μg of PCB 209 in 20 μl of hexane was added as surrogate. After the hexane fully evaporated, the slurry sample was extracted with 10 ml of acetone, followed by 10 ml of 1:1 acetone/hexane (v/v) twice, and 3 ml of hexane, respectively. The pooled extract was phase-separated with 8 ml of organic-free 2% NaCl solution. The obtained hexane layer was collected and concentrated to 2 ml. Tetrabutylammonium (TBA) sulfite was used to remove sulfur, following EPA Method 3660B. The TBA pretreated PCB extract was passed through a glass column (10 mm inner diameter) packed with 4 g Florisil and topped with 1.5 g anhydrous sodium sulfate. The column was pre-washed by 30 ml hexane. The eluate was loaded onto the column, followed by eluting with 30 ml n-hexane and 25 ml n-hexane/CH_2_Cl_2_ (4:1, v/v). The combined elutant was concentrated to exact 4.0 ml. The quantification of individual PCBs were performed by Hewlett Packard gas chromatograph (Model 6890) coupled with a micro-electron capture detector (μECD) and a 30-m DB-XLB capillary column (0.18 mm diameter and 0.18 μm film thickness; Agilent Technologies, Palo Alto, CA) (Xu et al., 2012, 2016). Complete dechlorination was confirmed by analyzing biphenyl in selected microcosms (duplicate bottles in each treatment at Week 36) at Energy & Environmental Research Center (EERC), University of North Dakota.

### DNA Extraction and Quantitative PCR

The sediment pellet samples from 0.5 ml of slurries were harvested as described previously (Xu et al., 2016). Total genomic DNA was extracted from the sediment pellets using the MoBio PowerSoil DNA Isolation Kit (MoBio, Carlsbad, CA) following the manufacturer’s protocols. Equal volumes of the triplicate DNA extracts at each sampling point were combined and mixed thoroughly for further microbial community analysis.

The 16S rRNA gene copies of total Bacteria, Fe(III)-reducing Family Geobacteraceae, *Dehalococcoides*, PCB degrading organism strains *ortho*-17 and *D. chlorocoercia* strain DF-1 (*o*-17/DF-1) were estimated using SYBR Green-based quantitative PCR (qPCR). Total Bacteria, Geobacteraceae*, Dehalococcoides*, and *o*-17/DF-1 were targeted with primer sets BAC338F/534R (Muyzer et al., 1993), Geo564F/840R(Cummings et al., 2003; Himmelheber et al., 2009), DHC1200F/1271R (He et al., 2003), and modified BAC908F/Dehal1265R (Edwards et al., 1989; Watts et al., 2005; Xu et al., 2012), respectively. qPCR reactions were performed in an ABI 7500 Real-Time PCR System (Applied Biosystems, Carlsbad, CA, United States) as described previously (Xu et al., 2016).

### Identification of Dechlorination Preferences

The dechlorination preferences were identified using a modified CPB analysis approach (Xu et al., 2016). Based on the known *ortho*, *meta*, and *para* chlorine positions, the neighboring conditions of each chlorine are considered. Totally, nine chlorine categories are defined, including unflanked *ortho* (UF *ortho*), flanked *ortho* (so called *meta*-flanked *ortho*), unflanked *meta* (UF *meta*), *ortho-*flanked *meta* (OF *meta*), *para*-flanked *meta* (PF *meta*), double-flanked *meta* (DF *meta*) (neighbored with one *ortho* and one *meta* chlorine), unflanked *para* (UF *para*), single-flanked *para* (SF *para*) (so-called *meta* flanked *para*), and double-flanked *para* (DF *para*).

## Results

### Headspace Gas

No O_2_ was detected in any of the microcosms. In the Hudson sediment microcosms amended with FeOOH (H-1-Fe and H-2-Fe), only a trace amount of CH_4_ (below 0.1% of the headspace gas) was detected in the time course of 51 weeks, indicating a complete inhibition of methanogenesis in these sediment microcosms. In the Grasse sediment microcosms, the addition of FeOOH significantly reduced but did not completely cease the generation of CH_4_ (see Supplementary Figure S1). The trends of CH_4_ production in different microcosm groups were generally G-2 > G-1 > G > G-2-Fe > G-1-Fe. The measured CH_4_ concentrations in FeOOH-amended Grasse microcosms were 30–40% lower than those in microcosms without added FeOOH. This suggests that FeOOH can moderately inhibit methanogenesis in the Grasse sediment. H_2_ was not detected in any Grasse sediment microcosms during the study period, while in the Hudson sediment microcosms, trace H_2_ (less than 0.2%) was only found at Week 0.

### Fe(III) Reduction

The reduction of Fe(III) was directly confirmed by tracking the concentrations of Fe(II) in the slurries over time. Due to the presence of indigenous Fe, Fe(II) concentrations in FeOOH-amended (H-1-Fe, H-2-Fe, G-1-Fe, and G-2-Fe), non-FeOOH-amended (H-1, H-2, G-2, and G-2), and no PCB control (H, G) microcosms were all measured to better elucidate the influences of supplementary FeOOH and/or PCBs on Fe(III) reduction. The trends of increasing Fe(II) were distinct in the Hudson and the Grasse sediments, but very similar in the same sediment spiked with different PCB *Mixtures* (see Supplementary Figure S2). In the Grasse sediment microcosms, after approximately 15–18 weeks of incubation, the differences of Fe(II) levels between G-1-Fe (91.3 ± 4.0 mmole/kg slurry at Week 15) and G-1 (51.4 ± 1.2 mmole/kg slurry at Week 15) or between G-2-Fe (105.4 ± 5.1 mmole/kg slurry at Week 18) and G-2 (61.3 ± 1.2 mmole/kg slurry at Week 18) remained at around 40 mmole/kg slurry until the end of 51 weeks. This indicates the complete reduction of supplementary FeOOH was accomplished within a 15–18 weeks’ incubation. Meanwhile, dissolved Fe(II) remained at 1.2–1.5 mmole/kg slurry, indicating the biogenic Fe(II) was mainly in the adsorbed form. In contrast, the Hudson sediment microcosms exhibited a much slower Fe(III) reduction. By the end of 51 weeks, Fe(II) concentrations in H-1-Fe, H-1, H-2-Fe, and H-2 were 47.3 ± 4.1 mmole/kg, 15.2 ± 0.9 mmole/kg, 47.1 ± 0.7 mmole/kg, and 13.2 ± 1.4 mmole/kg, respectively. The difference in corresponding microcosms in the presence and in the absence of supplementary FeOOH was smaller than 40 mmole/kg, suggesting FeOOH in H-1-Fe and H-2-Fe was not completely reduced to Fe(II) within the time course of 51 weeks. Dissolved Fe(II) ranged from 0.4 to 0.6 mmole/kg after 51 weeks of incubation. Additionally, the spiking of PCBs slightly favored Fe(III) reduction (G-1/G-2 with G, H-1/H-2 with H), especially in the first 21 weeks of incubation (Supplementary Figure S2), suggesting a complex microbial interaction in the presence of added alternative electron acceptors.

### PCBs in Sediment Microcosms

**FIGURE 1:**
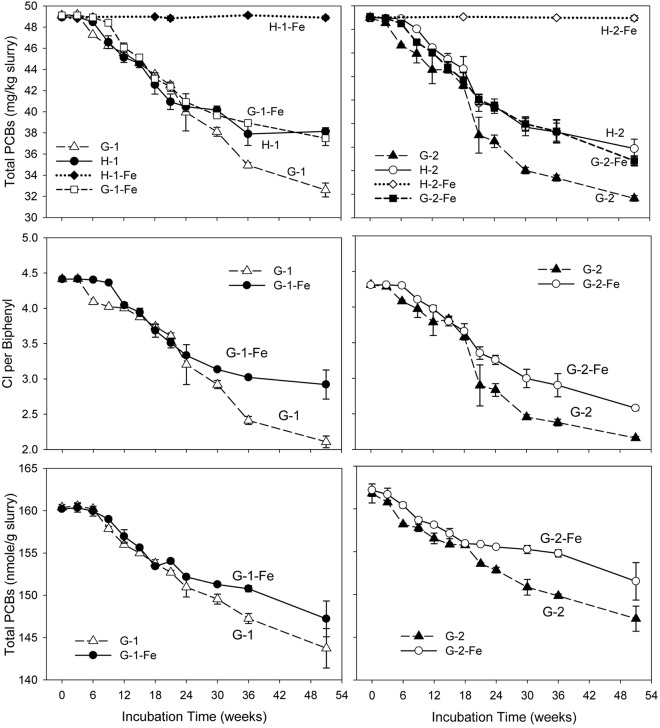
Total PCB mass concentrations, CPB values, and molar concentrations over time in sediment microcosms. H-1, Hudson sediment spiked with PCB *Mixture 1*; H-1-Fe, Hudson sediment spiked with PCB *Mixture 1* and FeOOH; G-1, Grasse sediment spiked with PCB *Mixture 1*; G-1-Fe, Grasse sediment spiked with PCB *Mixture 1* and FeOOH; H-2, Hudson sediment spiked PCB *Mixture 2*; H-2-Fe, Hudson sediment spiked PCB *Mixture 2* and FeOOH; G-2, Grasse sediment spiked with PCB *Mixture 2*; G-2-Fe, Grasse sediment spiked with PCB *Mixture 2* and FeOOH. Data plotted are averages of triplicates. Error bars represent standard deviation. Error bars not visible are smaller than the symbol size.

No dechlorination products were observed in sterile controls (data not shown) or the FeOOH-amended Hudson sediment microcosms within the 51 weeks’ incubation (Supplementary Tables S2, S3). This suggests that dechlorination was microbially catalyzed and the addition of FeOOH led to the complete inhibition of PCB dechlorination in the Hudson sediment. In addition, the negligible levels of generated CO_2_ and CH_4_ in H-1-Fe and H-2-Fe suggest low microbial activity in FeOOH-amended Hudson sediment microcosms. By contrast, supplementary FeOOH only moderately inhibited PCB dechlorination in the Grasse sediment microcosms. **Figure 1** depicts the total PCB mass over time for each experiment. Generally, the addition of FeOOH resulted in a longer lag time and lower dechlorination extent in the Grasse sediment microcosms. The lag time periods were as long as 6–9 weeks in G-1-Fe and approximately 6 weeks in G-2-Fe, which were around 3 weeks longer than lag times in G-1 and G-2. In addition, as mentioned earlier, it took 15–18 weeks to completely reduce the supplementary FeOOH in the Grasse sediment microcosms. PCB dechlorination occurred at least 9 weeks prior to the completion of FeOOH reduction, suggesting the concurrent Fe(III) reduction and PCB dechlorination. Comparing to the initially spiked PCBs of 50.0 mg/kg slurry, significantly higher residual PCBs (37.5 ± 0.7 and 36.8 ± 0.4 mg/kg slurry in G-1-Fe and G-2-Fe) were observed in FeOOH-amended sediment microcosms than those in non-FeOOH-amended sediment microcosms (32.6 ± 0.7 and 33.6 ± 0.2 mg/kg slurry in G-1 and G-2) after 51 weeks of incubation. Additionally, the reduction trends of total PCB mass were very similar in the Grasse sediment regardless of the PCB mixture spiked. The partial inhibition was also indicated by the changes of CPB values in the presence of supplementary FeOOH. By the end of 51 weeks, the CPB values for G-1-Fe and G-2-Fe declined by 33.8 and 40.2%, whereas the CPB values for the non-FeOOH-amended G-1 and G-2 declined by 52.2 and 49.9%. Seen in **Figure 1**, the declining trends of total PCB mass and CPB values in FeOOH-amended and non-FeOOH-amended Grasse sediment microcosms had not reached the plateau phase by the end of 51 weeks. This suggests the potential for further dechlorination in the Grasse sediment microcosms after even 1 year of incubation wasn’t changed by supplementary FeOOH.

Dechlorination cannot change the number of PCB molecules unless monochlorinated biphenyls are completely dechlorinated to biphenyl molecules. Excluding any experimental loss of PCBs, the decrease of total PCB moles is attributed to complete dechlorination. Illustrated in **Figure 1**, the reduction of total PCB molar concentrations in G-1-Fe and G-2-Fe was significant (*p* < 0.001), but less than that in non-FeOOH-amended G-1 and G-2. This was confirmed by biphenyl analysis (data not shown). Analysis of biphenyl suggested that the source of identified biphenyls was not the residual PAHs in sediments, but the spiked PCBs. The decrease of total PCB moles in G-1-Fe and G-2-Fe was mainly attributed to complete dechlorination of the only non-*ortho* congener spiked in the microcosms-PCB 12 (34-CB) (see Supplementary Tables S4, S5).

**FIGURE 2:**
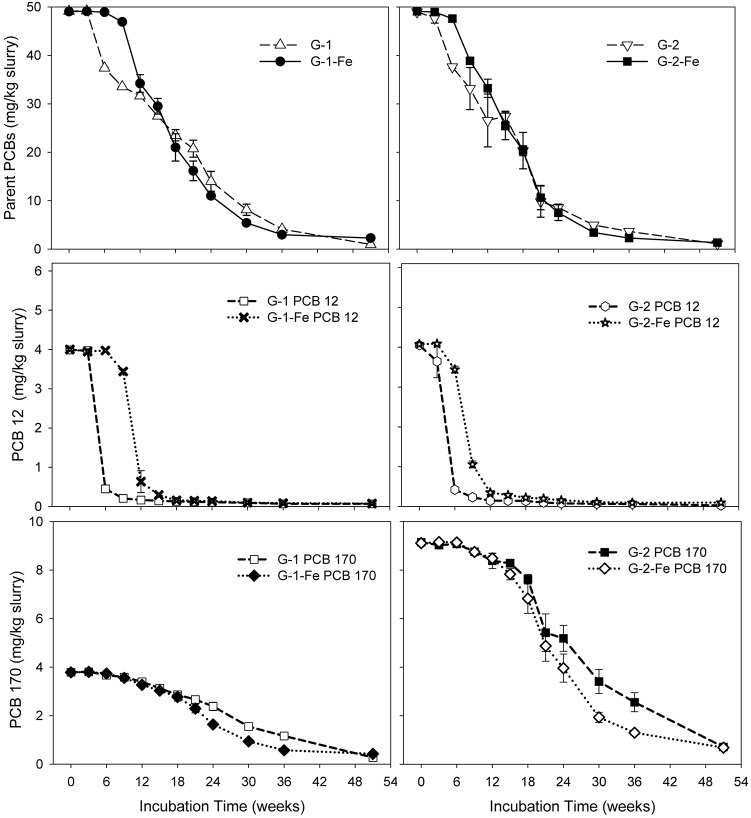
Mass concentrations of parent PCBs, PCB 12, and PCB 170 over time in sediment microcosms. Data plotted are averages of triplicates. Error bars represent standard deviation. Error bars not visible are smaller than the symbol size.

Although FeOOH moderately inhibited overall PCB dechlorination in the Grasse sediment microcosms, the influence of FeOOH on the first step dechlorination of spiked PCB congeners was complex. Seen in **Figure 2**, by the end of 51 weeks, over 96% of parent PCBs had been transformed in all the Grasse sediment microcosms. The less extensive dechlorination observed in G-1-Fe and G-2-Fe was not due to the lack of capability to remove the first chlorine atom from spiked PCB congeners. The changes of typical congeners PCB 12 and PCB 170 in Grasse sediment microcosms are illustrated in **Figure 2**, and the changes of other congeners are provided in Supplementary Figures S3–S6. Based on the relative amount of parent PCB congeners under methanogenic (G-1 and G-2) and Fe(III)-reducing (G-1-Fe and G-2-Fe) conditions, the parent PCB congeners were classified into three groups (**Table 2**). The first step of dechlorination for 10 out of 13 parent congeners in this study were either enhanced or not affected with FeOOH addition. Looking into the identical PCB congeners (PCB 5, 12, 64, and 71) spiked in the two PCB *Mixtures*, PCB *Mixture 2* tended to favor more rapid dechlorination of these congeners, as well as the concentration normalized PCB 170 (concentrations were normalized based on PCB 170 concentrations at Week 0) (**Figure 2** and Supplementary Figures S3, S4).

**Table 2:** Comparison of relative amounts of parent PCBs in the Grasse sediment microcosms.

Relative amount in treatments			PCB congeners	Duration
G-1/G-2	<	G-1-Fe/G-2-Fe	PCB 5(23-CB), PCB 12 (34-CB), PCB 71(26-34-CB)	3–51 weeks
G-1/G-2	=	G-1-Fe/G-2-Fe	PCB 64(236-4-CB), PCB 82(234-23-CB), PCB 97(245-23-CB), and PCB 99(245-24-CB)	15–51 weeks
G-1/G-2	>	G-1-Fe/G-2-Fe	PCB 105(234-34-CB), PCB 114(2345-4-CB), PCB 144(2346-25-CB), PCB 149(236-245-CB), PCB 153(245-245-CB), PCB 170(2345-234-CB)	18–36 weeks

*PCB, polychlorinated biphenyl.*

Notably, congener specific analysis of FeOOH-amended Grasse sediment microcosms revealed that *ortho* dechlorination occurred in G-1-Fe (Supplementary Table S4). The two identified pathways were PCB 25(24-3-CB) to PCB 13(3-4-CB) and PCB 28(24-4-CB) to PCB 15(4-4-CB). After 51 weeks of incubation, PCB 13 concentrations were found to be 1.2, 1.9, and 0.7 nmole/g slurry in triplicates, while the concentrations of PCB 15 were 2.3, 1.4, and 0.9 nmole/g slurry in triplicates. The above two *ortho* dechlorination pathways were also observed in non-FeOOH-amended G-1, suggesting the addition of FeOOH did not inhibit *ortho* dechlorination activity. Considering the changes of PCB 3(4-CB) in G-1-Fe, the concentrations were 10.8 ± 0.1 (10.8, 10.8, 10.9), 9.0 ± 0.6 (9.5, 8.2, 9.2), and 8.5 ± 4.6 (13.9, 5.4, 6.3) nmole/g at Week 18, Week 36, and Week 51, respectively. In other words, PCB 3 was reduced in two of the triplicates at Week 51, but increased sharply in the third. Moreover, the only spiked non-*ortho* PCB congener PCB 12(34-CB) was found to be at 0.7 ± 0.0, 0.4 ± 0.0, and 0.3 ± 0.0 nmole/g at Week 18, Week 36, and Week 51, respectively and could not contribute to the apparent increase of PCB 3 observed at Week 51. These results suggest that (1) dechlorination of unflanked *para* chlorines resumed after a long incubation; (2) the observed leveled PCB 3 in one of the microcosms at Week 51 was not directly linked to the dechlorination of PCB 12. The possible pathways included *meta/para* dechlorination of PCB 13(3-4-CB) or PCB 15(4-4-CB) and *ortho* dechlorination of PCB 7(24-CB) and PCB 8(2-4-CB). Regardless of the intermediates, the original parent PCB congeners were the dioxin-like mono-*ortho* PCB 105(234-34-CB) and PCB 114(2345-4-CB).

### Identification of Attacked Chlorines

**FIGURE 3:**
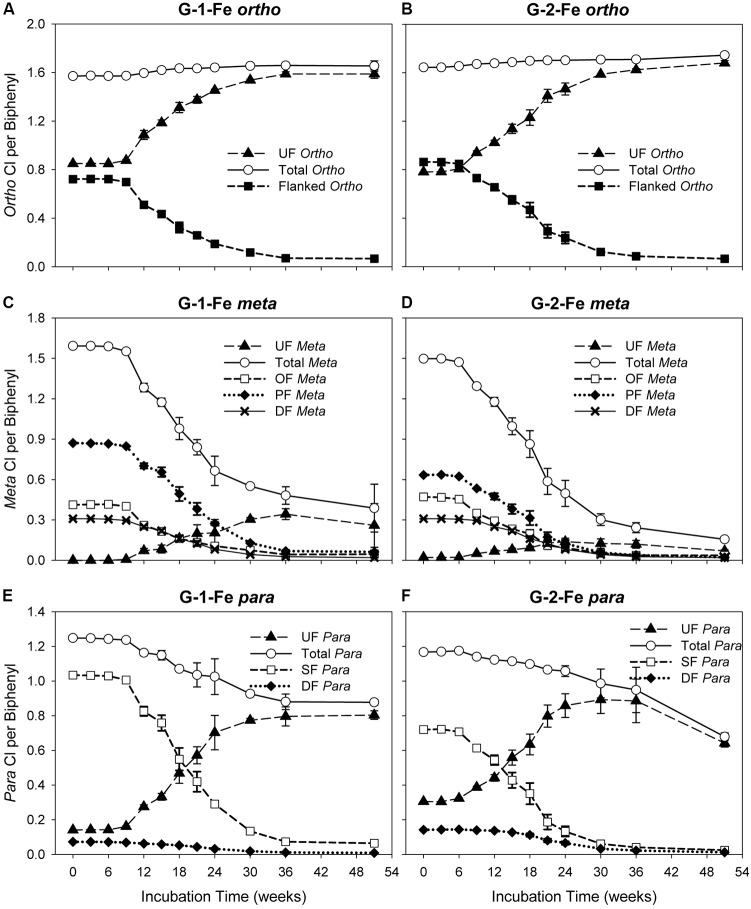
Changes of *ortho*, *meta*, *para* chlorines over time in G-1-Fe and G-2-Fe. **(A)** *ortho* in G-1-Fe; **(B)** *ortho* in G-2-Fe; **(C)** *meta* in G-1-Fe; **(D)** *meta* in G-2-Fe; **(E)** *para* in G-1-Fe; **(F)** *para* in G-2-Fe. UF, unflanked; SF, single-flanked; DF, double-flanked; OF, *ortho*-flanked; PF, *para*-flanked. All data points averaged triplicate microcosms. Error bars represent standard deviation. Error bars not visible are smaller than the symbol size.

The changes of *ortho* CPB (OCPB), *meta* CPB (MCPB), and *para* CPB (PCPB) for FeOOH-amended Grasse sediment microcosms are plotted in **Figure 3**. Illustrated in **Figures 3A,B**, OCPB values remained high in both G-1-Fe and G-2-Fe. In G-1-Fe, OCPB values slightly increased from 1.57 ± 0.00 to 1.66 ± 0.00 from Week 0 to Week 30. Thereafter, CPB values of 1.66 ± 0.01 and 1.65 ± 0.04 appeared at Week 36 and Week 51, respectively. In G-2-Fe, OCPB values kept increasing from 1.64 ± 0.00 to 1.74 ± 0.02 in the 51 weeks of incubation. The increase of OCPB was caused by the reduction of total molar concentration due to complete dechlorination of non-*ortho* PCB congener (PCB12, 34-CB) as decreasing the denominator in the OCPB calculation. A stable or reduced number of *ortho* chlorines was observed at Week 51 in G-1-Fe (**Figure 3A**), where *ortho* dechlorination occurred and was comparable with the reduction of total PCB molar concentration, resulting in similar decreases in the numerator and denominator in OCPB. In both G-1-Fe and G-2-Fe, unflanked *ortho* chlorines increased, whereas flanked *ortho* chlorines decreased over time. Considering the limited *ortho* dechlorination in G-1-Fe but not in G-2-Fe, the increase of unflanked *ortho* chlorines was due to the removal of *ortho-* or double-flanked *meta* chlorines, which resulted in fewer flanked chlorines on the *ortho* positioned chlorines but no overall change in the total number of *ortho* chlorines. By the end of 51 weeks, the remaining flanked *ortho* chlorines were as low as 0.07 ± 0.01 and 0.06 ± 0.00 in G-1-Fe and G-2-Fe, respectively, suggesting almost all *ortho* chlorines were unflanked at the end of the study. These findings were consistent with those observed in G-1 and G-2 (Xu et al., 2016), indicating the effect of FeOOH on the shifts of OCPBs was very limited.

*Meta* dechlorination was preferred but PCB composition dependent. The initial MCPB values for both mixtures were similar (see in **Figures 3C,D**). After 51 weeks of incubation, MCPB values declined from 1.59 ± 0.00 to 0.39 ± 0.18 in G-1-Fe and from 1.50 ± 0.00 to 0.16 ± 0.01 in G-2-Fe. Approximately 80–90% of *meta* chlorines were removed and the declining trends suggest a further *meta* chlorination potential. Compared with G-1 (0.16 ± 0.05 at Week 51) and G-2 (0.17 ± 0.01 at Week 51), the inhibitory effect of supplementary FeOOH was relatively limited. The *meta* dechlorination rates in the rapid phase of G-1-Fe (9–24 weeks) and G-2-Fe (6–30 weeks) were 0.056 ± 0.002 Cl/week and 0.051 ± 0.002 Cl/week, respectively, which exceeded the corresponding rapid phase *meta* dechlorination rates in G-1 (0.043 ± 0.004 Cl/week) and G-2 (0.043 ± 0.002 Cl/week). Seen in **Figures 3C,D**, unflanked *meta* chlorines increased in the first 30–36 weeks, then decreased slightly (solid triangle). Due to the lack of *ortho* removal before 36 weeks, the accumulated unflanked *meta* chlorines could be attributed to the dechlorination of single-/double-flanked *para* chlorines, where *ortho* chlorine was absent for the target phenyl ring. The maximum unflanked *meta* chlorines per biphenyl for G-1-Fe (0.34 ± 0.04 at Week 36) were much higher than those for G-1 (0.18 ± 0.01 at Week 30), indicating a partially inhibitory effect for the unflanked *meta* dechlorination. But in G-2-Fe and G-2, the maximum unflanked *meta* chlorines per biphenyl were similar (0.14 ± 0.03 for G-2-Fe; 0.13 ± 0.01 for G-2). Thereafter, the slightly decreased unflanked *meta* chlorines were the result of dechlorination of unflanked *meta* chlorines. Similarly, the declining of *ortho*-flanked *meta* chlorines (open square) was caused by removal of *meta* chlorines. By comparing the reduction rates of *ortho*-flanked *meta* chlorines and *meta*-flanked *ortho* chlorines in the rapid phase (9–24 weeks in G-1-Fe and 6–24 weeks in G-2-Fe), *meta*-flanked *ortho* chlorines (0.035 ± 0.002 Cl/week for G-1-Fe, 0.035 ± 0.001 Cl/week for G-2-Fe) decreased faster than *ortho*-flanked *meta* chlorines (0.021 ± 0.003 Cl/week for G-1-Fe; 0.021 ± 0.001 Cl/week for G-2-Fe), suggesting *meta* dechlorination attacked both *ortho*-flanked and double-flanked *meta* chlorines. Moreover, the shifts of the sub *meta* chlorine groups illustrated that overall *meta* dechlorination was mainly due to the reduction of *para*-flanked *meta* chlorines (solid diamond) rather than *ortho*-flanked *meta* or double-flanked *meta*. Also, this confirmed the *meta* dechlorination preference when *meta* chlorines were *para*-flanked. In general, *meta* dechlorination was prevalent in FeOOH-amended Grasse sediment microcosms, and *para*-flanked, *ortho*-flanked, and double-flanked *meta*, as well as relatively limited unflanked *meta*, were all targeted.

Compared to *meta* chlorination, *para* dechlorination was less extensive in FeOOH-amended Grasse sediment microcosms; approximately 30–40% of *para* chlorines were removed (**Figures 3C–F**). In G-1-Fe, from Week 9 to Week 36, averaged *para* dechlorination rate was 0.013 ± 0.001 Cl/week, while in G-2-Fe, from Week 6 to Week 36, the rate was 0.007 ± 0.000 Cl/week. From Week 36 to Week 51, PCBP values for G-1-Fe remained at approximately 0.88, whereas the values for G-2-Fe decreased from 0.88 ± 0.13 to 0.68 ± 0.02. Meanwhile, the unflanked *para* per biphenyl values remained at around 0.80 for G-1-Fe, and declined from 0.80 ± 0.02 to 0.64 ± 0.02 for G-2-Fe. Compared with non-FeOOH-amended G-1 and G-2, 75 and 80% of *para* chlorines were removed and the remaining unflanked *para* chlorines per biphenyl were only 0.32 ± 0.04 and 0.18 ± 0.01 after 51 weeks of incubation. These results suggest that (1) the remaining *para* chlorines were predominantly unflanked after a long time of incubation in G-1-Fe and G-2-Fe; (2) the inhibitory effect of FeOOH on PCB dechlorination was solely due to the loss of *para* dechlorinating activities targeting unflanked *para*. The unflanked *para* dechlorination resumed in G-2-Fe after 36 weeks of incubation but not in G-1-Fe, suggesting that PCB composition affected dechlorination preferences. Seen in **Figures 3E,F**, the reduction rates of single-flanked *para* chlorines (open square) (0.047 ± 0.001 Cl/week from Week 9 to Week 24 for G-1-Fe, 0.033 ± 0.001 Cl/week from Week 6 to Week 24 for G-2-Fe) were three to four times faster than the overall *para* removal rates shown above. Along with the reduction of single-flanked *para* chlorines, unflanked *para* (solid triangle) increased rapidly at rates 0.035 ± 0.001 Cl/week, 0.031 ± 0.002 Cl/week in the same time periods for G-1-Fe and G-2-Fe, respectively. This suggests *para* dechlorination preferred flanked *para* chlorines, but *meta* removal was more prevalent for PCBs with a single *meta* and *para* chlorine on the same phenyl ring. In addition, double-flanked *para* chlorines, although not dominant in either mixture decreased over time, where *para* and *meta* dechlorination both occurred.

### Quantification of Putative PCB Dechlorinating Bacteria and Fe(III)-Reducing Bacteria

**FIGURE 4:**
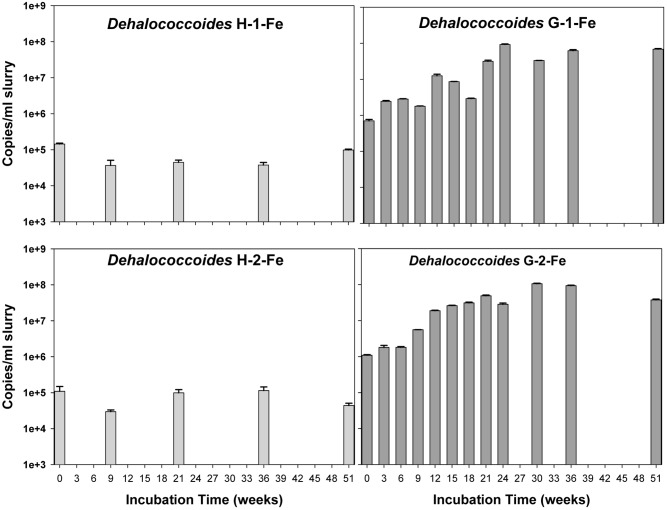
Quantitative assessment of *Dehalococcoides* 16S rRNA genes in H-1-Fe, G-1-Fe, and H-2-Fe, G-2-Fe. All data points averaged duplicate tests. Error bars represent standard deviation.

**FIGURE 5:**
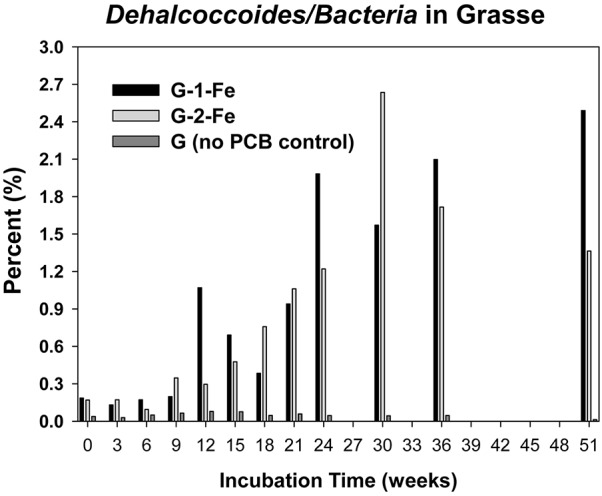
Relative abundance of *Dechalococcoides* 16S rRNA genes in total *Bacteria* 16S rRNA genes of G-1-Fe, G-2-Fe, and G.

The copy numbers of 16S rRNA genes of putative PCB dechlorinating bacteria *Dehalococcoides* over time are plotted in **Figure 4**. Initially, *Dehalococcoides* 16S rRNA genes in the Grasse sediment were one order of magnitude higher than those in the Hudson sediment. In H-1-Fe and H-2-Fe, no increase of *Dehalococcoides* 16S rRNA genes was observed over the course of incubation and the concentrations remained below 1.5 × 10^5^ copies/ml, which was the minimum *Dehalococcoides* 16S rRNA gene number found in H-1 and H-2 showing dechlorination activities. This suggests that the complete inhibition of PCB dechlorination in the FeOOH-amended Hudson sediment microcosms was very likely due to the lack of active *Dehalococcoides*. By contrast, G-1-Fe and G-2-Fe showed clear increasing trends of *Dehalococcoides* 16S rRNA genes over time. The *Dehalococcoides* 16S rRNA genes increased from 2.4 × 10^6^± 1.2 × 10^5^ to 9.2 × 10^7^ ± 3.1 × 10^6^ copies/ml in G-1-Fe and from 1.8 × 10^6^ ± 2.5 × 10^5^ to 1.1 × 10^8^ ± 3.1 × 10^6^ copies/ml in G-2-Fe. The supplementary FeOOH led to 5–10 times more *Dehalococcoides* 16S rRNA genes than observed in G-1 and G-2. The high *Dehalococcoides* 16S rRNA genes found at Week 51 indicate a potential of continuing dechlorination in G-1-Fe and G-2-Fe. The relative abundance of *Dehalococcoides* in G-1-Fe and G-2-Fe expressed as the averaged percentage of *Dehalococcoides* 16S rRNA genes in total *Bacteria* 16S rRNA genes is shown in **Figure 5**. The relative percentage of *Dehalococcoides* 16S rRNA genes increased from 0.2 to 2.5% in G-1-Fe and from 0.2 to 2.6% in G-2-Fe, while in G-1 and G-2, the percentage increased to around 1.0%. The addition of FeOOH enhanced not only the *Dehalococcoides* 16S rRNA gene numbers but also the relative abundance of *Dehalococcoides* in the microbial community. The *o*-17/DF-1 16S rRNA genes were much lower than those of *Dehalococcoides* and the relative abundance of *o*-17/DF-1 did not show any increase in G-1-Fe and G-2-Fe (data not shown). Thus, the *ortho* dechlorination activity observed in the Grasse sediment microcosms was likely associated with other unknown *ortho* dechlorinating organisms. Geobacteraceae 16S rRNA genes were from 2.5 × 10^5^± 4.8 × 10^4^ to 2.0 × 10^6^ ± 5.3 × 10^3^ copies/ml in H-1-Fe and H-2-Fe, while significantly higher Geobacteraceae 16S rRNA genes (5.5 × 10^5^± 1.3 × 10^4^ to 1.1 × 10^7^ ± 4.3 × 10^5^ copies/ml) were found in control group H (*p* < 0.01). Additionally, Geobacteraceae 16S rRNA genes in H-1-Fe/H-2-Fe had no significant differences with those in H-1/H-2 (*p* > 0.05). These indicate that there was no Geobacteraceae enrichment by the addition of FeOOH. The low Geobacteraceae level could explain the weak Fe(III) reduction observed in H-1-Fe and H-2-Fe. Meanwhile, Geobacteraceae 16S rRNA genes detected in the Grasse sediment microcosms remained one to three orders of magnitude higher than those in the Hudson sediment microcosms (data not shown). Notably, the addition of FeOOH did not further stimulate the growth of Geobacteraceae.

### Supplementary Carbon Sources and *Ortho* Dechlorination Enhancement

In the Hudson sediment microcosms amended with FeOOH, supplementary acetate or the fatty acid mixture led to extensive methanogenesis after the second addition at Week 36 (Supplementary Table S6). Along with the generation of CH_4_, enhanced reduction of Fe(III) was observed in carbon-amended microcosms at Week 36 and Week 51 for H-1-Fe and at Week 51 only for the fatty acid mixture amended H-2-Fe (Supplementary Figure S7). Notably, PCB dechlorination was observed after the addition of carbon sources. **Table 3** shows the mass concentrations of total parent PCB concentrations at Week 36 and Week 51. By the end of 51 weeks, total parent PCBs were reduced by 3–20% on the average of triplicate microcosms with two carbon sources additions (incubating with carbon sources from Week 27 to Week 51). The fatty acid mixture and sole acetate additions both reinitiated PCB dechlorination in H-1-Fe and H-2-Fe. The dominant pathways included PCB 5(23-CB) to PCB 1(2-CB), PCB 12(34-CB) to PCB 2(3-CB) and PCB 3(4-CB), and PCB 64(236-4-CB) to PCB 32(26-4-CB). Flanked *meta* and flanked *para* dechlorination took place first. However, 16S rRNA genes of *Dehalococcoides* and *o*-17/DF-1 did not increase apparently in H-1-Fe and H-2-Fe when PCB dechlorination occurred after the supplementation of carbon sources, indicating the existence of other PCB dechlorinating organisms not closely related to *Dehalococcoides* or *o*-17/DF-1 (data not shown). Additionally, it was possible that biogenic Fe(II) also induced abiotic dechlorination. In contrast, the additions of carbon sources did not facilitate further dechlorination in H-1 or H-2 (Supplementary Table S7).

**Table 3:** Total parent PCB concentrations (mg/kg slurry) of the FeOOH-amended sediment microcosms with/without additional carbon sources.

Sediment	Incubation Time	PCB *Mixture 1*			PCB *Mixture 2*		
		No Carbon	Acetate	Fatty Acid Mix	No Carbon	Acetate	Fatty Acid Mix
Hudson	36 weeks	49.1 ± 0.1^a^	49.1 ± 0.3	48.9 ± 0.2	49.0 ± 0.1	49.1 ± 0.1	45.9 ± 5.3^b^
	51 weeks	48.9 ± 0.2	42.3 ± 4.3	46.8 ± 0.1	49.0 ± 0.2	47.6 ± 0.3	40.2 ± 11.1^b^
Grasse	36 weeks	2.97 ± 0.41	2.10 ± 0.13	2.10 ± 0.04	2.27 ± 0.19	2.00 ± 0.22	2.08 ± 0.15
	51 weeks	2.28 ± 0.41	0.98 ± 0.03	1.00 ± 0.07	1.34 ± 0.05	1.08 ± 0.07	1.12 ± 0.06

*^a^Data were present by mean ± standard deviation. ^b^Dechlorination was very prevalent in one of the triplicate microcosms. PCB, polychlorinated biphenyl.*

The supplementation of carbon sources into FeOOH-amended Grasse sediment microcosms did not significantly enhance overall dechlorination by re-initiating unflanked *para* dechlorination activity. However, it did favor the reduction of parent PCBs (**Table 3**). The reduction was more apparent for the highly chlorinated parent PCB congeners, indicating a relatively high carbon source-dependence for these congeners. Surprisingly, rare *ortho* dechlorination, targeting PCB congeners with exclusive unflanked *ortho* chlorines, was apparently enhanced by the addition of acetate or the fatty acid mixture in both G-1 and G-1-Fe (congener-specific concentrations provided in Supplementary Tables S8, S9). *Ortho* dechlorination products, which were only detected at Week 51 in G-1 and G-1-Fe without additional carbon sources, appeared 15 weeks ahead with carbon source addition. The enhancement was relatively weak in G-1 (see Supplementary Material). By contrast, in the triplicate acetate/fatty acid mixture amended G-1-Fe microcosms, PCB 13 and PCB 15 concentrations increased sharply from Week 36 to Week 51 (PCB 13 and PCB 15 were 15.0 ± 1.2, 7.0 ± 1.2 for acetate-amended G-1-Fe, 12.7 ± 5.0, 10.1 ± 5.0 for fatty acid mixture-amended G-1-Fe at Week 51) and no significant difference of the concentrations of *ortho* dechlorination products was observed by adding two carbon sources (Supplementary Table S9). Moreover, a rough mass balance could be obtained by the transformation of PCB 25 to PCB 13 and PCB 28 to PCB 15 between Week 36 and Week 51, where PCB 105, PCB 114, and their first and second generation dechlorination products were very low (see text in Supplementary Material and Supplementary Table S9). By the end of the incubation, in the G-1-Fe microcosms with carbon sources, *ortho* dechlorination products PCB 13 and PCB 15 accounted for 10–15% of the total PCB molar, which were over five times higher than those in the regular G-1-Fe microcosms without extra carbon sources. These findings suggest the similar enhancement ability of acetate or the fatty acid mixture and further confirmed the existence of *ortho* dechlorination pathways in Grasse River sediment. However, the 16S rRNA gene numbers of *Dehalococcoides* and *o*-17/DF-1 in the microcosms with carbon sources did not change significantly comparing with the microcosms without carbon source addition (data not shown). In addition, Fe(II) analysis in the FeOOH-amended Grasse sediment microcosms with supplementary carbon sources showed that no further Fe(III) reduction was induced. This further confirmed that Fe(III) reduction had completed prior to the addition of carbon sources. The supplementation of carbon sources stimulated prevalent methane production. The fatty acid mixture induced more methane production than did the addition of acetate alone (Supplementary Figure S1 and Supplementary Table S6).

## Discussion

### Impact of Indigenous Sediment Properties and PCB Composition Under Fe(III)-Reducing Condition

Many studies suggested that the dechlorination of PCBs was controlled by their sediment geochemical properties and/or microbial species (Wiegel and Wu, 2000; Yan et al., 2006; Park et al., 2011; Payne et al., 2012). This is the first observation of complete dechlorination inhibition in Fe(III)-amended Hudson sediment. A previous study had reported that 50 mM FeOOH reduced dechlorination by 12% in enriched cultures of sediment collected from a different location in the Hudson River (Morris et al., 1992). Due to the lack of sediment property characterization in that study, the difference is difficult to explain. The relatively low background Fe concentration (approximately 19 mmole/kg slurry), low Geobacteraceae and slow Fe(III) reduction observed in our Hudson sediment suggest poor acclimation to high Fe concentration, resulting in complete inhibition of PCB dechlorination and methanogenesis in FeOOH-amended Hudson sediment microcosms. In the Fe(III)-amended Grasse sediment microcosms, there was a 20–30% reduction of overall dechlorination in a time course of 51 weeks compared to the non-FeOOH-amended Grasse sediment microcosms, mainly due to less *para* dechlorination. The high background Fe content (approximately 64 mmole/kg slurry), abundant Geobacteraceae and fast Fe(III) reduction suggest good capability for reducing competing electron acceptor and easy resumption of PCB dechlorination. Additionally, our previous study showed that *para* dechlorination in the high Fe content Grasse sediment (G-1, G-2) was slower than that in the low Fe content Hudson sediment (H-1, H-2) in the rapid phase (Xu et al., 2016). These results suggest that indigenous Fe content could profoundly influence dechlorination preferences. Also, FeOOH further slowed down *para* dechlorination, but facilitated *meta* dechlorination in the rapid phase of G-1-Fe and G-2-Fe, indicating that *meta*/*para* dechlorination activities might be competing with each other and Fe(III) was likely a controlling factor. Moreover, PCB composition, even with four identical PCB congeners, the same total concentrations, and similar chlorine contents, was found to significantly affect PCB dechlorination in FeOOH-amended Grasse sediment microcosms. PCB *Mixture 2* favored the dechlorination of PCB 5, 12, 64, 71, and 170. This trend was found in non-FeOOH-amended G-1 and G-2, suggesting the addition of FeOOH did not alter the composition related dechlorination preferences. These findings might be explained by co-metabolism or halopriming effect in a mixture (Krumins et al., 2009; Fagervold et al., 2011; Xu et al., 2016). FeOOH, especially freshly precipitated FeOOH, has high specific surface area and thus can absorb significant amount of pollutants in sediment and soil (Herbich, 2000; Okkenhaug et al., 2013). In this study, freshly prepared FeOOH was introduced directly into the sediment microcosms and the microcosms were incubated statically. Therefore, it was likely that PCBs adsorbed on the sediment particles aggregated on FeOOH. Along with the reduction of Fe(III), there may have been more bioavailable PCBs in the niches, where prevalent Fe(III)-reducing bacteria as well as related PCB dechlorinators were expected. In addition, biogenic Fe(II), mostly as adsorbed Fe(II), was likely to contribute to PCB dechlorination (Watson et al., 2002; Gong et al., 2016; Liu et al., 2017). This hypothesis was partially supported by the observation that more hydrophobic PCB congeners (highly chlorinated PCB 144, 149, 153, 170, and coplanar PCB 105, 114) exhibited more rapid dechlorination when FeOOH was added. A previous study demonstrated enhanced PCB dechlorination following Processes M and Q with the addition of FeSO_4_ in Hudson River sediment and proposed the beneficial effect caused by precipitation of toxic sulfide by Fe^2+^ (Zwiernik et al., 1998). However, the dechlorination induced by FeS could not be excluded (Watson et al., 2002). FeCl_2_ without sulfate was also found to favor PCB dechlorination (Zwiernik et al., 1998). This further suggested that Fe(II) might be a reductant for chemical dechlorination. Generally, the mineral reactivity of dechlorination of chlorinated solvents was disorder FeS > FeS > FeS_2_ > sorbed Fe^2+^ (He et al., 2015). This indicates that the influences of Fe(II)-containing minerals on PCB dechlorination might be complicated.

### Putative PCB Dechlorinators and Fe(III) Reducers

Although exhibiting less extensive dechlorination, FeOOH-amended Grasse sediment microcosms showed an increase of *Dehalococcoides* 16S rRNA genes when compared with non-FeOOH-amended Grasse sediment microcosms. This indicates complex interactions between Fe(III)-reduction and *Dehalococcoides* growth. The possible mechanistic explanation is that some Fe(III) reducers within the Genus *Geobacter* of the Family Geobacteraceae, like *Geobacter sulfurreducens*, are involved in H_2_ generation in cooperation with hydrogen-oxidizing microorganisms or produce H_2_ via fermentation. H_2_ is believed to be the preferred electron donor for *Dehalococcoides* (Cord-Ruwisch et al., 1998; He et al., 2003, 2005; Wei and Finneran, 2011; Zanaroli et al., 2012b; Karatas et al., 2014). Enrichment of *Dehalococcoides* has been reported during dechlorination of chlorinated solvents under Fe(III)-reducing conditions (Wei and Finneran, 2011). Moreover, two known Fe(III)-reducing bacteria, *Geobacter thiogenes* strain K and *Geobacter lovleyi* strain SZ were capable of utilizing organohalides as their terminal electron acceptors for energy conservation and growth (Sung et al., 2006), and more recently, indirect evidence linking PCB dechlorination with Geobacteraceae was reported, although *D. mccartyi* was still considered as the main dechlorinators (Praveckova et al., 2016). These reports suggest complex dechlorination mechanisms in the presence of Fe(III) in addition to the potential for competition for electron donors. Geobacteraceae were not stimulated in Fe(III)-amended Grasse sediments, suggesting that electron acceptor Fe(III) was no longer the limiting factor in the Fe rich sediment. Similar findings were reported in Fe rich sediment from South China (Liu et al., 2017).

### Carbon Source and *Ortho* Dechlorination Enhancement

A previous study demonstrated that repeated addition of fatty acids (acetate, propionate, butyrate, and hexanoic acid) stimulated PCB dechlorination in carbon-limiting Hudson sediment, but not in high carbon content New Bedford Harbor and Silver Lake sediments.(Alder et al., 1993) In the present study, the influences of auxiliary carbon sources on PCB dechlorination were also sediment dependent. For the relatively low carbon and low Fe Hudson sediment, the addition of acetate or the fatty acid mixture did not enhance the overall dechlorination in H-1 and H-2 (under methanogenic conditions). This might be due to the experimental dechlorination limit observed as a flat plateau phase after 30 weeks. On the other hand, the addition of carbon sources led to the resumption of PCB dechlorination in FeOOH-amended microcosms (H-1-Fe and H-2-Fe, no methanogenesis activity before carbon sources addition), suggesting the effective compensation of the extra “electron donor demand” derived from Fe(III) (Wei and Finneran, 2011). For the relatively higher carbon content and high Fe Grasse sediment, the addition of acetate or the fatty acid mixture did not enhance the overall dechlorination rate and extent in all the sediment microcosms (G-1, G-2, G-1-Fe, and G-2-Fe), where methanogenesis was remained. However, auxiliary carbon sources apparently shortened the lag time for *ortho* dechlorination and greatly favored *ortho* removal in G-1-Fe. These results indicate that the impact of carbon source was not only controlled by the sediment background carbon content, but also by the other geochemical properties, such as Fe(III).

This work provides the first observation of *ortho* dechlorination enhancement by auxiliary carbon sources (acetate or a fatty acid mixture). The isolated *ortho* dechlorinators *o*-17 is known as an acetate-utilizing bacterium (Cutter et al., 2001; May et al., 2006; Fagervold et al., 2011). It is expected that the *ortho* dechlorinating bacteria in this study are also utilizing acetate as their electron donor and carbon source. As there was no significant difference for the enhancement of *ortho* removal in acetate-amended and the fatty acid mixture-amended microcosms, acetate was not likely the obligate carbon source in this Grasse sediment. Most previous studies observed *ortho* dechlorination capable of dechlorinating PCBs with two or more *ortho* chlorines (Vandort and Bedard, 1991; Pulliam Holoman et al., 1998; Cutter et al., 2001; May et al., 2006; Fagervold et al., 2011; Kjellerup et al., 2014). *O*-17, isolated from Baltimore Harbor marine sediment preferentially dechlorinates *meta*-flanked *ortho* chlorine when the *meta* chlorine is only *ortho*-flanked and the target PCB congener contains two to three *ortho* chlorines, such as PCB 65(2356-CB) and PCB 90(235-24-CB) (May et al., 2006; Fagervold et al., 2007, 2011). Although PCB 65 was ultimately dechlorinated to PCB 14(35-CB) with PCB 23(235-CB) as an intermediate, the *ortho* dechlorination activity was not sustained when only PCB 23 was spiked, indicating *o*-17 lacks *ortho* dechlorination activity for mono-*ortho* substituted PCBs (May et al., 2006). Another marine sediment from Hunters Point California, in which a fatty acid mixture of acetate, propionate and butyrate was added, exhibited *ortho* removal targeting PCB 116(23456-CB) (*meta* chlorines are double-flanked) and the only dechlorination product was PCB 14(35-CB), indicating a stepwise reduction of two *ortho* chlorines.(Kjellerup et al., 2014) *Ortho* dechlorination observed in two freshwater sediments (Silver Lake and Woods Pond) without auxiliary carbon source, attacked unflanked *ortho* chlorine on PCB 30(246-CB) (Williams, 1994). Our previous study also revealed unflanked *ortho* dechlorination activity on PCB 28(24-4-CB) and PCB 25(24-3-CB) in Grasse River sediment without auxiliary carbon source (Xu et al., 2016). In the present study, the single unflanked *ortho* chlorine removal activity was maintained under Fe(III)-reducing conditions despite no change in known *ortho* dechlorinating organism populations. This further supports the existence of distinct *ortho* dechlorinating organisms in Grasse River sediment. Also, an easy approach to selectively enhance *ortho* dechlorination was provided. Most recently, a study in Grasse River sediment microcosms focusing on Aroclor 1254 detoxification used dioxin-like PCB 105 as a test congener, in which PCB 28 and PCB 25 were also the dominant dechlorination products after 180 days (Kaya et al., 2017). Therefore, the *ortho* dechlorination preference with the potential for complete dechlorination of mono-*ortho* PCB congeners, including some dioxin-like congeners, might be crucial for further potential risk reduction in Grasse sediment.

## Conclusion

The amendment with FeOOH greatly affected PCB dechlorination and the influences were largely controlled by the indigenous differences in sediment. Generally, the low carbon content and low Fe Hudson sediment showed complete inhibition for PCB dechlorination after adding FeOOH, while the relatively high carbon content and high Fe Grasse sediment showed moderate inhibition in the presence of FeOOH. The dechlorination preferences analysis revealed that *para*-flanked *meta* dechlorination was primarily preferred followed by *ortho-*/double-flanked *meta* dechlorination and single-/double-flanked *para* dechlorination in Grasse sediment and the partially inhibitory effect was caused by the loss of unflanked *para* removal activity. Despite the longer lag time observed in the FeOOH-amended Grasse sediment microcosms, Fe(III) reduction and PCB reduction were found to take place concurrently. Rare *ortho* dechlorination pathways were confirmed in FeOOH-amended Grasse sediment microcosms. Auxiliary carbon sources (acetate, or a fatty acid mixture with acetate, propionate and butyrate) could reinitiate dechlorination in FeOOH-amended Hudson sediment microcosms, as well as support the favored *ortho* dechlorination in FeOOH-amended Grasse microcosms, indicating the utilization of acetate and/or fatty acids for *ortho* dechlorination related microorganisms in Grasse sediment.

## Author Contributions

YX has conducted the experiments, analyzed data, and prepared the manuscript. KG has written the manuscript. JV has conceived and led the project and finalized the manuscript. All authors approved the current version of the manuscript.

## Conflict of Interest Statement

The authors declare that the research was conducted in the absence of any commercial or financial relationships that could be construed as a potential conflict of interest.
