# Supplementary material for: Effects of Ferric Oxyhydroxide on Anaerobic Microbial Dechlorination of Polychlorinated Biphenyls in Hudson and Grasse River Sediment Microcosms: Dechlorination Extent, Preferences, Ortho Removal, and Its Enhancement
Source: Front Microbiol. 2018 Jul 20;9:1574. doi: 10.3389/fmicb.2018.01574 (PMC6062599; doi:10.3389/fmicb.2018.01574)
Supplement: Supplementary file 1 [file Presentation_1.PDF]

Effects of Ferric Oxyhydroxide on Anaerobic Microbial Dechlorination of Polychlorinated Biphenyls in Hudson and Grasse River Sediment Microcosms: Dechlorination Extent, Preferences, *Ortho* Removal and Its Enhancement

Yan Xu[12], Kelvin B Gregory[2], Jeanne M. VanBriesen[2*]*

Submitted to: *Frontiers in Microbiology*

* Corresponding author present address: Department of Municipal Engineering, School of Civil Engineering, Southeast University, Nanjing, Jiangsu, China, 210096.; phone: 86(25)83790757; fax: 86(25)83790757; e-mail: xuxucalmm@seu.edu.cn; Department of Civil and Environmental Engineering, Carnegie Mellon University, 5000 Forbes Avenue, Pittsburgh, PA 15213-3890 ; e-mail: jeanne@andrew.cmu.edu.

[1]Present address: Department of Municipal Engineering, School of Civil Engineering, Southeast University, Nanjing, Jiangsu, China, 210096.

[2] Present address: Department of Civil and Environmental Engineering, Carnegie Mellon University, 5000 Forbes Avenue, Pittsburgh, PA 15213-3890.

E-mail addresses: xuxucalmm@seu.edu.cn (Yan Xu), kgregory@andrew.cmu.edu (Kelvin Gregory), jeanne@andrew.cmu.edu (Jeanne VanBriesen)

Supplementary Material

Figure S1. Headspace methane production over time in the Grasse sediment microcosms

Figure S2. Fe(II) concentrations over time

Figure S3. Concentrations of PCB 5 over 51 weeks of incubation

Figure S4. Concentrations of PCB 64, 71 over 51 weeks of incubation

Figure S5. Concentrations of PCB 105, 114, 149 and 153 over 51 weeks of incubation

Figure S6. Concentrations of PCB 82, 97, 99 and 144 over 51 weeks of incubation

Figure S7. Total Fe(II) concentrations after 30 weeks of incubation with and without supplementary carbon sources

Table S1. Spiked PCB congener concentrations in sediment microcosms

Table S2. PCB congener concentrations in H-1-Fe at Week 0, 18, 21, 36 and 51

Table S3. PCB congener concentrations in H-2-Fe at Week 0, 18, 36 and 51

Table S4. Averaged PCB congener concentrations in the Grasse sediment microcosms spiked with PCB *Mixture 1* at Week 0, 18, 36 and 51

Table S5. Averaged PCB congener concentrations in the Grasse sediment microcosms spiked with PCB *Mixture 2* at Week 0, 18, 36 and 51

Table S6. Methane production (mmole/kg slurry) in FeOOH-amended sediment microcosms after additions of carbon sources

Table S7. Total PCB mass concentrations (mg/kg slurry) of the microcosms with and without supplementary carbon sources at Week 36 and Week 51

Table S8. PCB congener concentrations in acetate and the fatty acid mixture amended G-1 at Week 36 and 51 (nmole/g)

Table S9. PCB congener concentrations in acetate and the fatty acid mixture amended G-1-Fe at Week 36 and 51 (nmole/g)

Table S1. Spiked individual PCB congener concentrations in sediment microcosms

IUPAC#	Structure	PCB *Mixture 1*		PCB *Mixture 2*	
		mg/kg slurry	nmole/g slurry	mg/kg slurry	nmole/g slurry
PCB 5	23	5.77	25.9	5.77	25.9
PCB 12	34	4.23	19.0	4.23	19.0
PCB 64	236-4	6.31	21.6	6.31	21.6
PCB 71	26-34	3.69	12.6	3.69	12.6
PCB 82	234-23	/	/	3.53	10.8
PCB 97	245-23	/	/	6.78	20.8
PCB 99	245-24	/	/	9.69	29.7
PCB 105	234-34	9.36	28.7	/	/
PCB 114	2345-4	0.64	2.0	/	/
PCB 144	2346-25	/		1.25	3.5
PCB 149	236-245	7.78	21.6	/	/
PCB 153	245-245	8.52	23.6	/	/
PCB 170	2345-234	3.71	9.4	8.75	22.1
**Total PCBs**		**50.0**	**164.3**	**50.0**	**165.9**

Two PCB *Mixtures* contained 13 PCB congeners. PCB 5 (23-CB)/PCB 12 (34-CB) and PCB 64 (236-4-CB)/PCB 71(26-34-CB) had identical concentrations in both PCB *Mixtures*. PCB 170 (2345-234-CB) had distinct concentrations in the two PCB *Mixtures*. *Mixture 2* contained greater than twice of PCB 170 than *Mixture 1*.

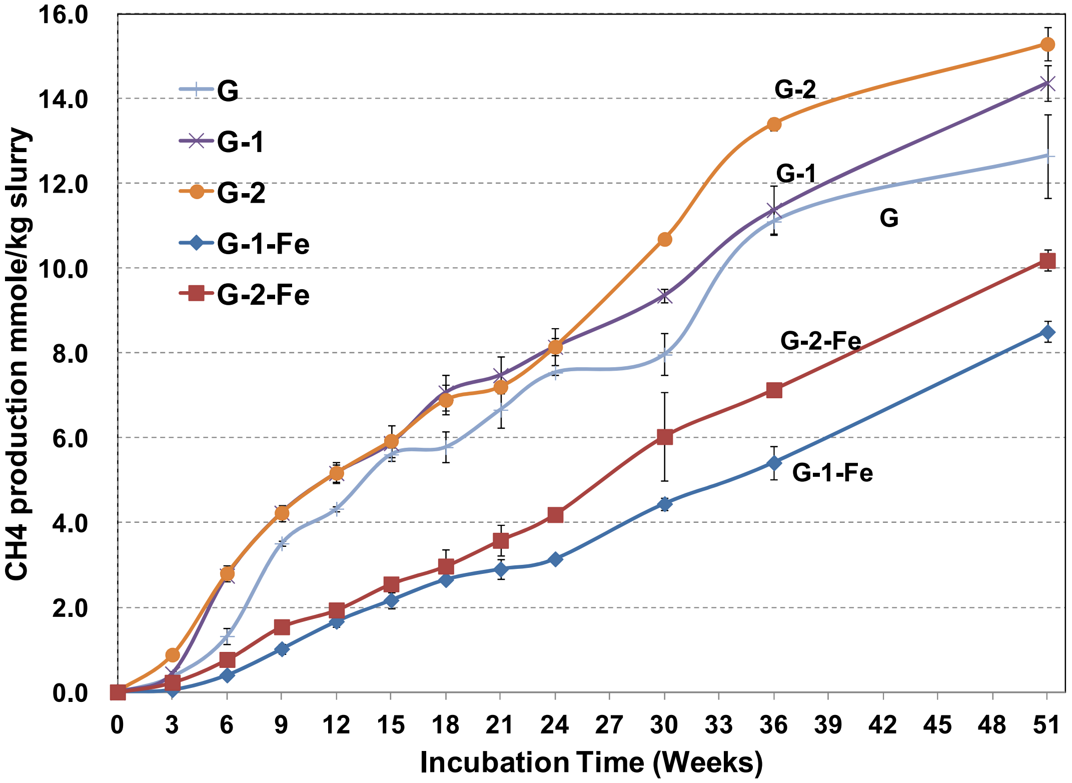

Figure S1. **Headspace methane production over time in the Grasse sediment microcosms**

All data points averaged replicate microcosms. Error bars represent standard deviation, with some error bars smaller than the symbol size.

A

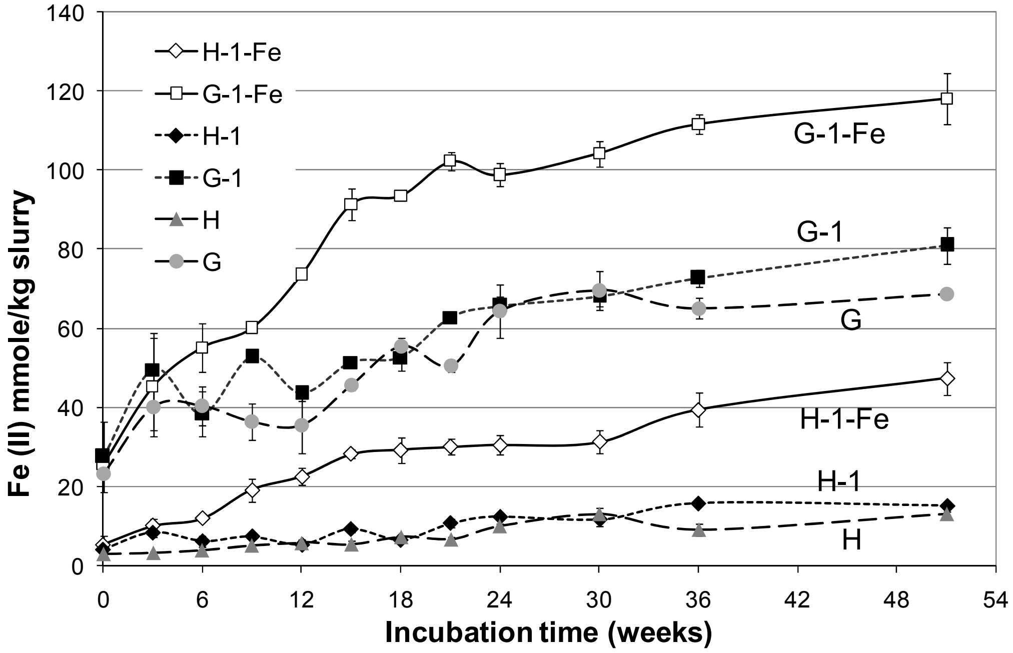

B

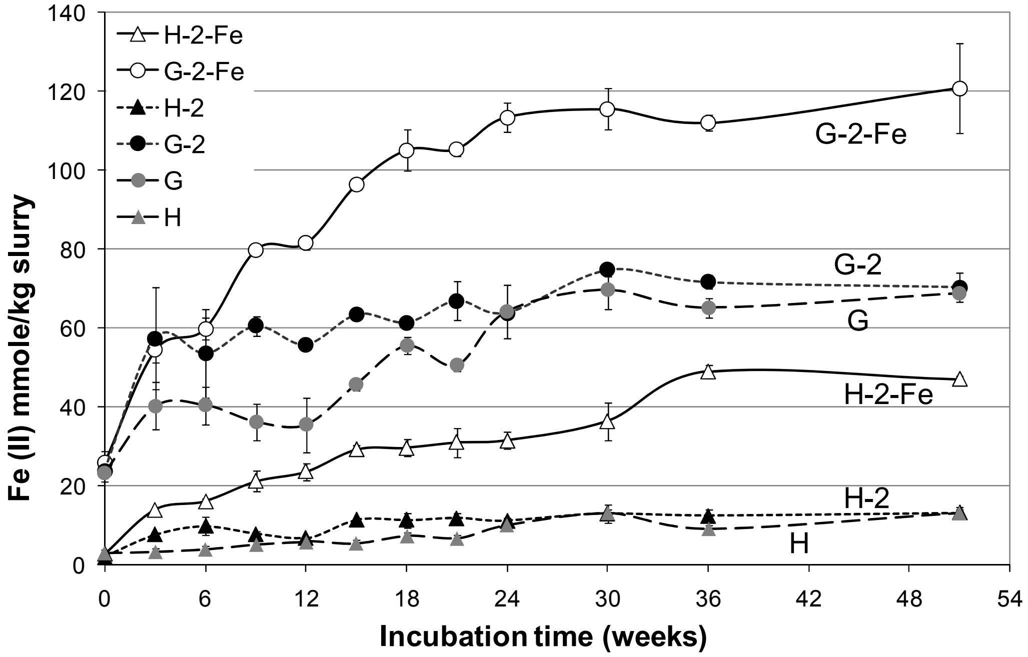

Figure S2. Fe(II) concentrations over time. (A) Microcosms spiked with PCB *Mixture* 1 and no PCB controls; (B) Microcosms spiked with PCB *Mixture 2* and no PCB controls.

All data points averaged replicate microcosms. Error bars represent standard deviation, with some error bars smaller than the symbol size.

Table S2. PCB congener concentrations in H-1-Fe at Week 0, 18, 21, 36 and 51

IUPAC	Concs (mg/kg)	H-1-Fe (mg/kg)				
		0 W	18 W	21 W	36 W	51 W
PCB 5	5.77	5.30 ± 0.04	5.38 ± 0.01	5.34 ± 0.04	5.29 ± 0.08	5.26 ± 0.08
PCB 12	4.23	3.94 ± 0.02	3.95 ± 0.06	3.93 ± 0.08	3.92 ± 0.09	3.87 ± 0.10
PCB 64	6.31	6.01 ± 0.01	6.11 ± 0.08	6.05 ± 0.09	6.06 ± 0.03	6.09 ± 0.06
PCB 71	3.69	3.67 ± 0.04	3.60 ± 0.04	3.55 ± 0.03	3.60 ± 0.03	3.52 ± 0.06
PCB 105	9.36	9.28 ± 0.01	9.23 ± 0.02	9.17 ± 0.05	9.36 ± 0.05	9.21 ± 0.08
PCB 114	0.64	0.68 ± 0.01	0.68 ± 0.01	0.69 ± 0.01	0.70 ± 0.01	0.69 ± 0.01
PCB 149	7.78	7.80 ± 0.04	7.65 ± 0.10	7.73 ± 0.02	7.77 ± 0.04	7.79 ± 0.04
PCB 153	8.52	8.63 ± 0.01	8.58 ± 0.06	8.57 ± 0.10	8.63 ± 0.05	8.68 ± 0.05
PCB 170	3.71	3.82 ± 0.01	3.81 ± 0.02	3.80 ± 0.01	3.79 ± 0.03	3.77 ± 0.03
Total PCBs	50.0	49.13 ± 0.06	48.98 ± 0.08	48.84 ± 0.22	49.12 ± 0.14	48.89 ± 0.18

Table S3. PCB congener concentrations in H-2-Fe at Week 0, 18, 36 and 51

IUPAC	Concs (mg/kg)	H-2-Fe (mg/kg)			
		0 W	18 W	36 W	51 W
PCB 5	5.77	5.39 ± 0.09	5.39 ± 0.05	5.32 ± 0.05	5.25 ± 0.03
PCB 12	4.23	4.01 ± 0.11	4.04 ± 0.09	3.98 ± 0.04	3.91 ± 0.02
PCB 64	6.31	6.02 ± 0.04	6.03 ± 0.06	6.10 ± 0.04	6.14 ± 0.11
PCB 71	3.69	3.56 ± 0.04	3.63 ± 0.03	3.61 ± 0.05	3.60 ± 0.02
PCB 82	3.53	3.43 ± 0.02	3.49 ± 0.02	3.40 ± 0.02	3.42 ± 0.01
PCB 97	6.78	6.69 ± 0.06	6.71 ± 0.02	6.77 ± 0.01	6.67 ± 0.09
PCB 99	9.69	9.43 ± 0.09	9.51 ± 0.09	9.53 ± 0.05	9.54 ± 0.08
PCB 144	1.25	1.28 ± 0.01	1.27 ± 0.03	1.28 ± 0.01	1.27 ± 0.00
PCB 170	8.75	9.19 ± 0.08	9.02 ± 0.11	9.01 ± 0.07	9.15 ± 0.04
Total PCBs	50.0	49.00 ± 0.23	49.07 ± 0.09	48.99 ± 0.08	48.96 ± 0.20

Table S4. Averaged PCB congener concentrations in the Grasse sediment microcosms spiked with PCB *Mixture 1* at Week 0, 18, 36 and 51

IUPAC#*	Structure	**G-1-Fe** (nmole/g)				**G-1** (nmole/g)			
		0 W	18 W	36 W	51 W	0 W	18 W	36 W	51 W
PCB 1	2-	0	23.2	24.3	25.1	0	23.6	42.4	29.3
PCB 2	3-	0	0	0	0	0	0	0	0
PCB 3	4-	0	10.8	9.0	8.5	0	11.3	5	**16.8**
PCB 4	2-2	0	0	0	2.2	0	0.4	14.9	19.5
PCB 5	23-	24.0	0.8	0.3	0.3	23.9	0.4	0.2	0.1
PCB 6	2-3	0	1.3	2.3	1.6	0	2.2	5.7	3.4
PCB 7	24-	0	0.1	0.2	3.2	0	0.1	0.1	0.3
PCB 8	2-4	0	0.5	0.7	0.7	0	1.8	2.1	0.6
PCB 9	25-	0	0.2	0.3	0.0	0	0.2	0	0
PCB 10	26-	0	0	0	0.1	0	0.9	21.9	27
PCB 12	34-	17.9	0.7	0.4	0.3	17.9	0.6	0.3	0.3
**PCB 13**	**3-4**	0	0	0	**1.3**	0	0	0	**0.6**
**PCB 15**	**4-4**	0	0	0	**1.5**	0	0	0	**3.9**
PCB 17	24-2	0	0.1	0.3	2.2	0	0.3	1.2	1.2
PCB 18	25-2	0	0.1	0.6	0.6	0	0.3	2.3	1.6
PCB 19	26-2	0	0	0	0.8	0	0.5	11.9	18.5
PCB 20	23-3	0	0.3	0	0	0	0.3	0	0
PCB 22	23-4	0	0.1	0	0	0	0.1	0	0
PCB 23	235-	0	0	0	0	0	0	0.2	0
PCB 24	236-	0	0	0	0.1	0	0	0	0
PCB 25	24-3	0	9.9	18.9	13.2	0	3.2	0.6	1.1
PCB 27	26-3	0	0.2	2.4	1.4	0	1.4	5.2	0.9
PCB 28	24-4	0	3.9	5.5	3.3	0	4	0.6	0.3
PCB 31	25-4	0	0.1	0.2	0.2	0	0.2	0.7	0.1
PCB 32	26-4	0	16.8	27.5	28.5	0	15.9	4.3	3.5
PCB 33	34-2	0	0.5	0	0	0	0.3	0	0
PCB 42	23-24	0	0	0.0	0.1	0	0.1	0	0.1
PCB 43	235-2	0	0	0.1	0.2	0	0	0.4	0.1
PCB 44	23-25	0	0.1	0.0	0.0	0	0	0	0
PCB 47	24-24	0	4.7	11.9	12.0	0	4	3.7	4.4
PCB 49	24-25	0	1.9	7.5	4.9	0	1.2	1.3	1.4
PCB 50	246-2	0	0.0	0.1	0.0	0	0	0	0.1
PCB 51	24-26	0	3.6	17.0	17.1	0	3	2.7	1.8
PCB 52	25-25	0	3.8	7.3	5.2	0	1.5	2.1	2.5

PCB 53	25-26	0	0.2	1.6	0.6	0	0.2	2.3	0.7
PCB 55	234-3	0	0.2	0	0	0	0.3	0	0
PCB 56	23-34	0	0.3	0	0	0	1	0	0
PCB 60	234-4	0	0	0	0	0	0.1	0	0
PCB 61	2345-	0	0.2	0.1	0.1	0	0.1	0.1	0.1
PCB 63	235-4	0	0.7	0.7	0.8	0	0.5	0.6	0.2
PCB 64	236-4	20.5	5.1	0.4	0.2	20.8	5.9	0.4	0.2
PCB 66	24-34	0	2.1	0.1	0.2	0	1.7	0.2	0.1
PCB 71	26-34	12.4	10.3	1.9	1.8	12.5	8.2	0.7	0.2
PCB 74	245-4	0	0.1	0	0	0	0.1	0	0
PCB 83	235-23	0	0.2	0.1	0.1	0	0.2	0.2	0
PCB 90	235-24	0	0.8	2.2	2.6	0	0.6	1.4	0.5
PCB 91	236-24	0	0.4	0.2	0.5	0	0.3	0.3	0.1
PCB 95	236-25	0	0.1	0.0	0.0	0	0.2	0.2	0
PCB 99	245-24	0	0.7	0.2	0.4	0	0.5	0.2	0.1
PCB 101	245-25	0	0.8	0.1	0.1	0	0.9	0.2	0
PCB 102	245-26	0	1.9	0.5	0.8	0	1	0.7	0
PCB 105	234-34	28.3	9.3	0.8	0.4	28.3	13.6	1.2	0.3
PCB 114	2345-4	2.1	0.8	0.1	0.1	2.1	1	0.2	0.1
PCB 129	2345-23	0	0	0	0	0	0	0	0
PCB 130	234-235	0.0	0.2	0.1	0.2	0	0.2	0.1	0
PCB 137	2345-24	0	0.1	0.1	0.1	0	0.1	0.1	0.1
PCB 138	234-245	0	0.1	0	0.1	0	0	0	0
PCB 149	236-245	21.5	15.3	2.2	1.7	21.6	16.2	3.4	0.4
PCB 153	245-245	23.9	12.7	1.2	1.0	23.7	15.8	2.4	0.6
PCB 170	2345-234	9.6	7.0	1.5	1.1	9.6	7.2	2.9	0.7
Total Amount		**160.2**	**153.4**	**150.8**	**147.2**	**160.4**	**153.8**	**147.3**	**143.7**

Note: PCB 4 and 10 were coeluted, but with a retention time difference of 0.1 min, therefore a shoulder peak was obtained and split by the shoulder detection function of ChemStation software. Bold values indicate *ortho* dechlorination products.

* PCB congeners listed were those detectable in at least one microcosm.

Table S5. Averaged PCB congener concentrations in the Grasse sediment microcosms spiked with PCB *Mixture 2* at Week 0, 18, 36 and 51

IUPAC#	Structure	**G-2-Fe** (nmole/kg)				**G-2** (nmole/kg)			
		0 W	18 W	36 W	51 W	0 W	18 W	36 W	51 W
PCB 1	2-	0	23.3	24.0	23.6	0	24	24.2	22.6
PCB 2	3-	0	0.0	0.0	0.0	0	0	0	0
PCB 3	4-	0	12.3	11.7	8.8	0	12.1	6.8	6.3
PCB 4	2-2	0	0.2	13.9	44.8	0	6.2	58.9	65.6
PCB 5	23-	24.7	0.8	0.2	0.2	24.6	0.3	0.2	0.2
PCB 10	26-	0	0.0	0.2	0.5	0	3.7	21.6	25.1
PCB 12	34-	18.3	1.0	0.4	0.4	18.2	0.6	0.3	0.1
PCB 13	3-4	0	0	0	0	0	0	0	0
PCB 15	4-4	0	0	0	0	0	0	0	0
PCB 16	23-2	0	0.2	0.0	0.0	0	0.2	0	0
PCB 17	24-2	0	7.8	17.4	5.2	0	6.8	1.6	1.5
PCB 18	25-2	0	1.7	3.1	0.6	0	2.7	1.7	2
PCB 19	26-2	0	0.0	0.4	1.3	0	0	1.7	2.3
PCB 27	26-3	0	0.3	0.8	0.9	0	3.4	8.4	5.1
PCB 32	26-4	0	17.5	29.8	30.3	0	14.2	1.2	1.4
PCB 40	23-23	0	0.6	0.0	0.0	0	0.4	0	0
PCB 42	23-24	0	6.1	0.7	0.3	0	3.3	0.4	0.2
PCB 43	235-2	0	0.0	0.1	0.2	0	0.1	1	0.5
PCB 44	23-25	0	1.8	0.2	0.0	0	1.1	0.1	0
PCB 47	24-24	0	14.5	31.1	21.0	0	10.8	5.3	5
PCB 48	245-2	0	0.1	0.0	0.0	0	0.6	0	0
PCB 49	24-25	0	5.3	7.9	3.7	0	3.4	1.3	2.2
PCB 50	246-2	0	0.0	0.4	1.1	0	0.1	0.3	0.2
PCB 53	25-26	0	0.0	0.2	0.0	0	0.2	0.8	0.5
PCB 64	236-4	20.7	5.6	0.3	0.2	20.5	3.6	0.4	0.2
PCB 71	26-34	12.1	9.0	0.9	0.3	12.2	7.5	0.5	0.1
PCB 82	234-23	10.5	3.7	0.2	0.2	10.4	3.6	0.3	0.1
PCB 83	235-23	0	0.2	0.1	0.0	0	0.2	0.4	0.2
PCB 90	235-24	0	1.3	3.4	3.9	0	1.2	2.8	2.9
PCB 95	236-25	0	0.0	0.0	0.0	0	0.1	0	0
PCB 97	245-23	20.6	9.1	0.4	0.3	20.4	9.2	0.6	0.2
PCB 99	245-24	28.8	12.3	0.7	0.6	28.9	13.2	1.1	0.3
PCB 103	246-25	0	1.7	2.5	1.1	0	1.1	0.5	0.4
PCB 129	2345-23	0	0.1	0.0	0.0	0	0.1	0.1	0

PCB 130	234-235	0	0.3	0.1	0.1	0	0.2	0.2	0.1
PCB 137	2345-24	0	0.2	0.2	0.1	0	0.2	0.2	0.1
PCB 138	234-245	0	0.1	0.1	0.0	0	0.1	0.1	0
PCB 144	2346-25	3.5	1.8	0.1	0.1	3.5	2	0.2	0.1
PCB 170	2345-234	23.1	17.3	3.3	1.7	23.1	19.3	6.5	1.8
Total Amount		**162.2**	**155.9**	**154.8**	**151.5**	**161.8**	**155.8**	**149.8**	**147.2**

* PCB congeners listed were those detectable in at least one microcosm.

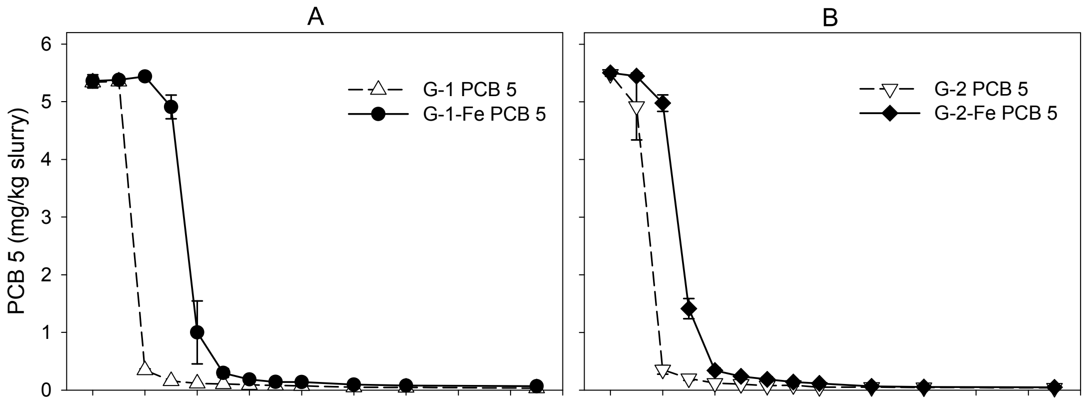

Figure S3. Concentrations of PCB 5 over 51 weeks of incubation.

Data plotted are averages of triplicates. Error bars represent standard deviation; Error bars not visible are smaller than the symbol size.

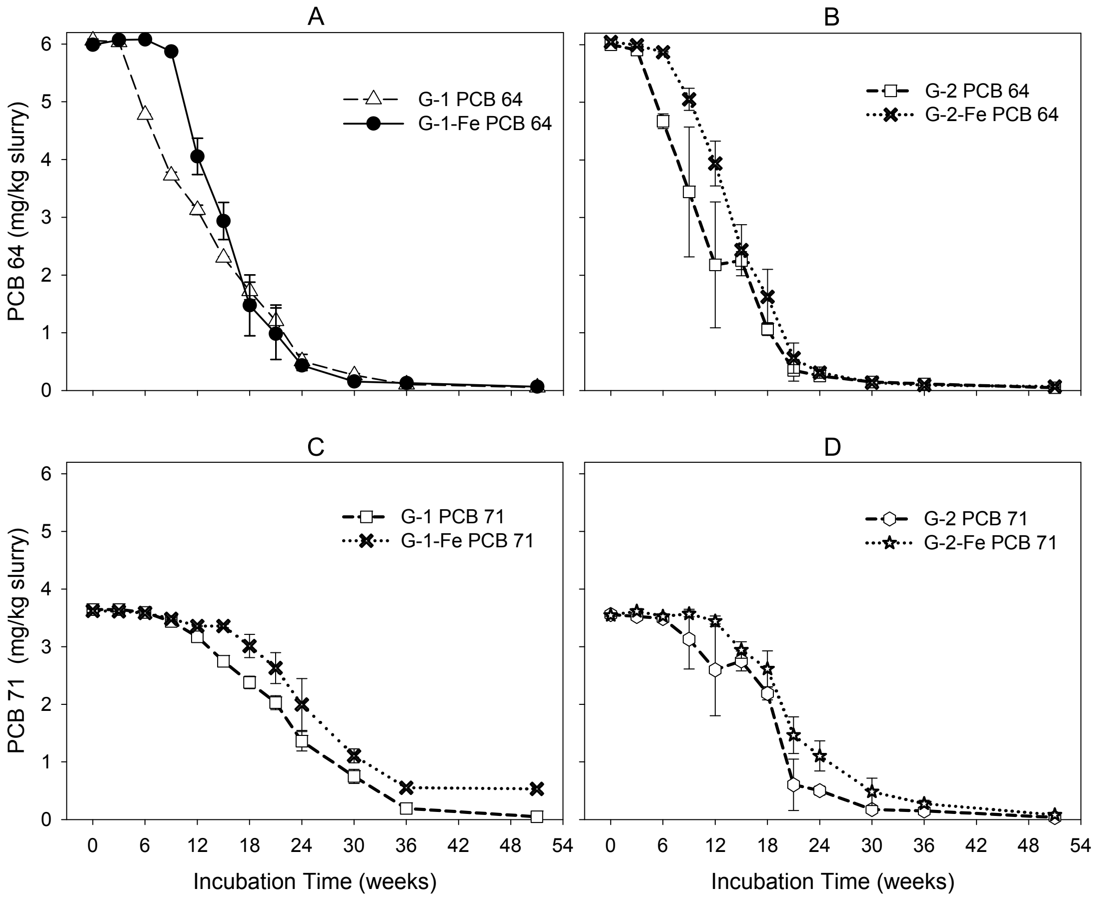

Figure S4. Concentrations of PCB 64, 71 over 51 weeks of incubation.

Data plotted are averages of triplicates. Error bars represent standard deviation; Error bars not visible are smaller than the symbol size.

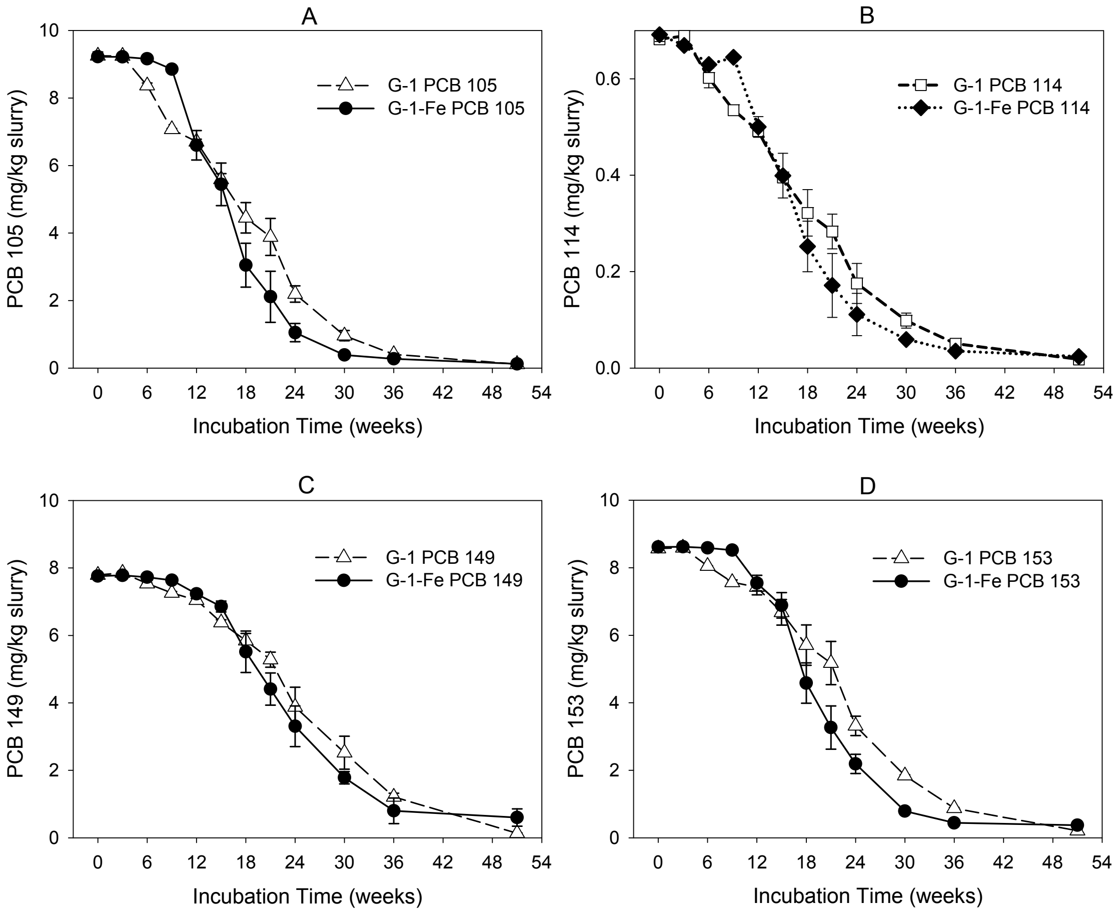

Figure S5. Concentrations of PCB 105, 114, 149 and 153 over 51 weeks of incubation.

Data plotted are averages of triplicates. Error bars represent standard deviation; Error bars not visible are smaller than the symbol size.

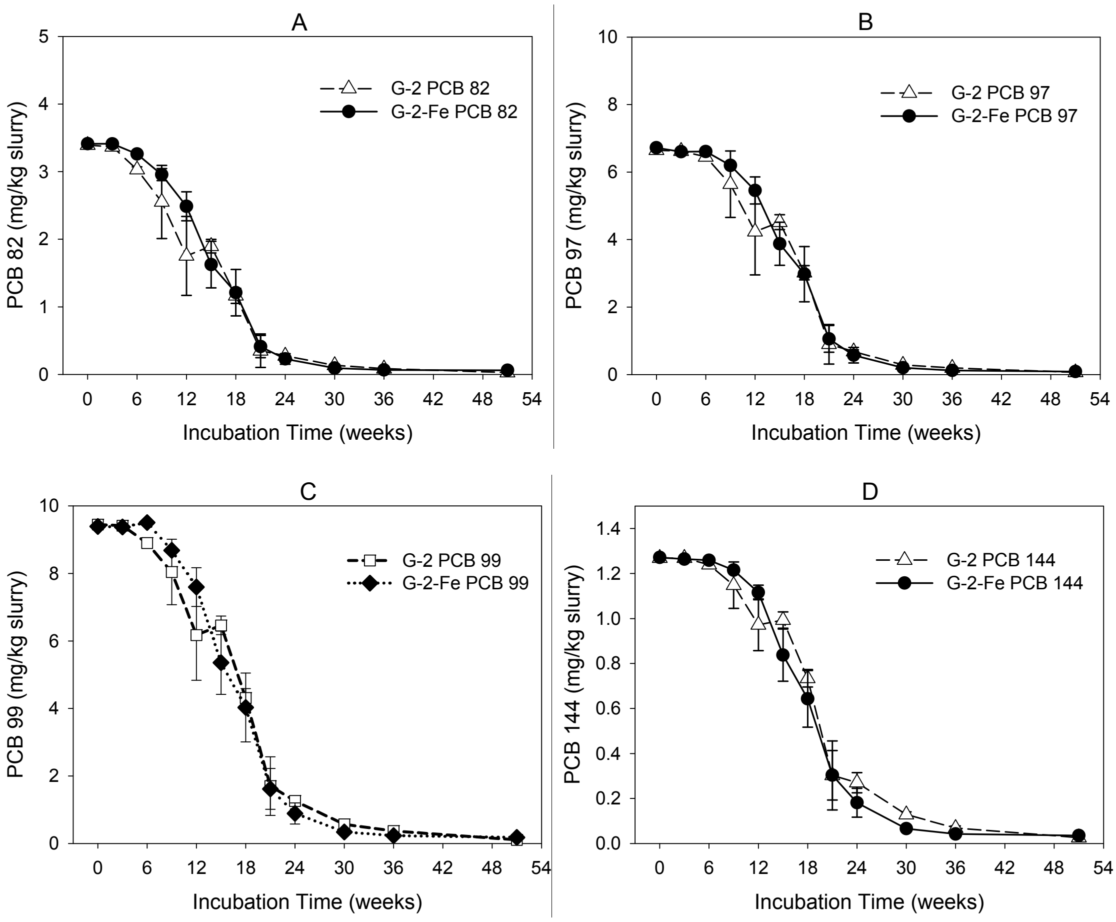

Figure S6. Concentrations of PCB 82, 97, 99 and 144 over 51 weeks of incubation.

Data plotted are averages of triplicates. Error bars represent standard deviation; Error bars not visible are smaller than the symbol size.

Table S6. Methane production (mmole/kg slurry) in FeOOH-amended sediment microcosms after additions of carbon sources.

Sediments	Incubation Time	PCB *Mixture 1*		PCB *Mixture 2*	
		Acetate	Fatty Acids	Acetate	Fatty Acids
Hudson	30 weeks	0.01±0.00[a]	0.00±0.00	0.04±0.05	0.01±0.00
	36 weeks	0.50±0.40	0.04±0.02	4.08±2.69	0.78±0.90
	51 weeks	41.78±0.61	33.91±27.04[b]	36.42±3.68	60.84±69.22[b]
Grasse	30 weeks	5.92±0.56	6.28±0.19	6.05±0.28	6.68±0.10
	36 weeks	17.71±0.81	39.01±1.31	19.04±0.18	43.49±1.08
	51 weeks	26.66±0.72	51.57±1.39	26.35±0.38	49.17±1.39

[a] Data were present by mean±standard deviation. [b] Methane productions were diverged in three replicates.

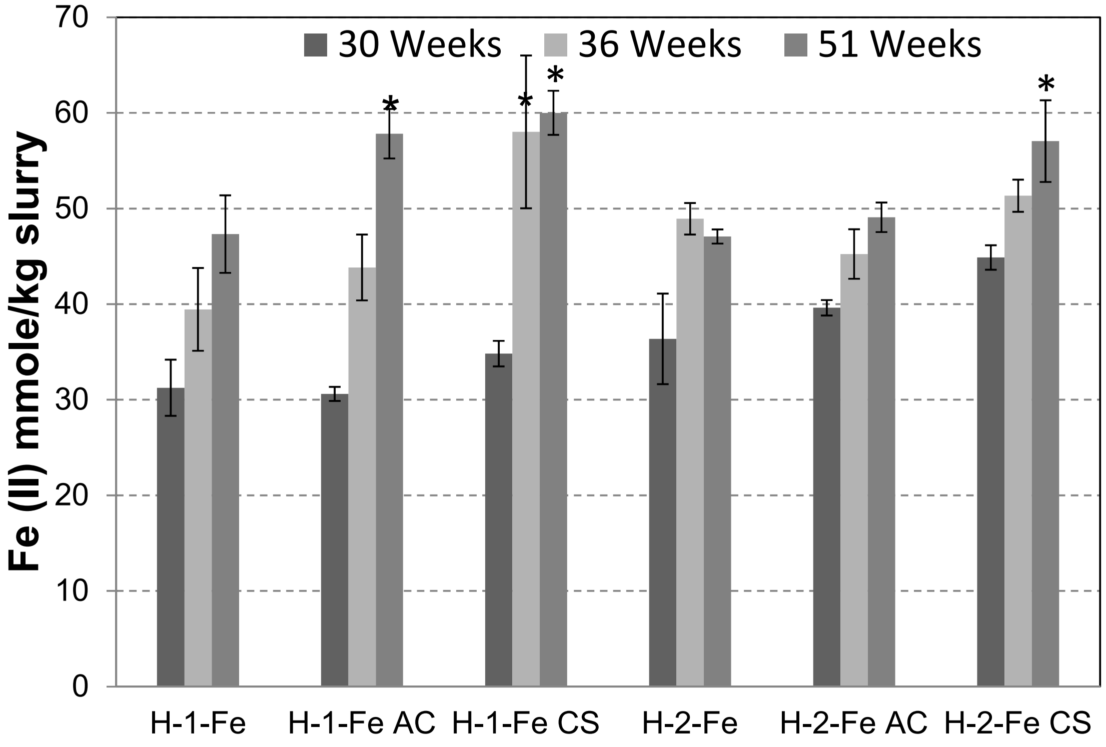

Figure S7. Total Fe(II) concentrations after 30 weeks of incubation with and without supplementary carbon sources.

AC: acetate; CS: acetate, propionate and butyrate mixture. * Significant difference between non-carbon-amended and carbon-amended groups (p<0.05).

Table S7. Total PCB mass concentrations (mg/kg slurry) of the microcosms with and without supplementary carbon sources at Week 36 and Week 51

Sediments		Incubation Time	PCB *Mixture 1*			PCB *Mixture 2*		
			No Carbon	Acetate	Fatty Acids	No Carbon	Acetate	Fatty Acids
**Hudson**		36 weeks	37.9 ± 1.1	39.1 ± 1.2	39.2 ± 0.6	39.3 ± 0.8	39.7 ± 0.4	39.2 ± 0.4
		51 weeks	38.1 ± 0.4	38.0 ± 1.1	38.3 ± 0.6	37.9 ± 0.8	38.8 ± 0.4	37.9 ± 0.7
	**Fe**	36 weeks	49.1 ± 0.1	49.1 ± 0.3	48.9 ± 0.2	49.0 ± 0.1	49.1 ± 0.1	48.3 ± 1.1
	**Fe**	51 weeks	48.9 ± 0.2	46.7 ± 0.6	47.7 ± 0.1	49.0 ± 0.2	48.4 ± 0.2	46.5 ± 2.1
**Grasse**		36 weeks	34.9 ± 0.3	35.2 ± 0.1	35.5 ± 1.1	35.4 ± 0.3	35.4 ± 0.1	35.2 ± 0.3
		51 weeks	32.6 ± 0.7	33.8 ± 0.4	34.1 ± 1.2	33.6 ± 0.2	34.1 ± 0.3	34.1 ± 0.1
	**Fe**	36 weeks	38.9 ± 0.1	38.7 ± 0.2	38.8 ± 0.0	39.3 ± 1.0	39.7 ± 0.1	39.5 ± 0.5
	**Fe**	51 weeks	37.5 ± 0.7	37.2 ± 0.2	36.8 ± 0.1	36.8 ± 0.4	38.7 ± 0.2	38.0 ± 0.5

Table S8. PCB congener concentrations in acetate and the fatty acid mixture amended G-1 at Week 36 and 51 (nmole/g)

IUPAC#	Structure	G-1 AC Week 36			G-1 AC Week 51			G-1 CS Week 36			G-1 CS Week 51		
		A	B	C	A	B	C	D	E	F	D	E	F
1	2-	39.4	41.5	43.1	34.7	37.3	38.1	38.1	31.2	45.3	33.7	29.4	40.2
2	3-	0	0	0	0	0	0	0	0	0	0	0	0
3	4-	8.8	9.3	10.2	9.2	8.4	11.8	8.3	10.5	7.3	10.4	12.2	6.4
4	2-2	14.0	15.0	14.6	14.9	15.3	15.4	11.4	7.3	18.9	12.6	10.5	18.8
5	23-	0.2	0.2	0.2	0.2	0.2	0.1	0.2	0.3	0.2	0.2	0.2	0.2
6	2-3	4.2	3.3	1.7	4.0	2.7	1.5	1.5	3	1.4	1.6	1.9	1.5
7	24-	0.3	0.7	1.0	0.3	0.4	0.3	0.3	0.6	0.4	0.4	0.2	0.4
8	2-4	1.6	2.7	2.6	1.8	2.9	3.2	3.2	6.8	2.1	3.9	7.0	2.4
9	25-	0.3	0.1	0.0	0.0	0.0	0.0	0	0	0	0	0	0
10	26-	23.0	21.1	20.7	23.3	21.0	20.6	16.5	2.9	20.7	16.7	3.3	20.5
12	34-	0.3	0.3	0.4	0.2	0.3	0.3	0.4	0.4	0.3	0.3	0.4	0.3
13	3-4	0.7	0.5	0.9	2.2	1.7	1.6	0.9	0.8	0	1.8	1.9	0.7
15	4-4	1.4	1.8	1.8	2.4	2.2	2.9	4.2	5.6	0.4	5.3	7.0	1.0
16	23-2	0	0	0	0	0	0	0	0	0	0	0	0
17	24-2	1.7	2.7	3.4	1.9	3.3	3.9	2.4	2.9	1.4	2.6	2.7	1.4
18	25-2	1.4	0.6	0.4	1.2	0.6	0.4	0.5	0.3	0.4	0.5	0.4	0.5
19	26-2	14.0	13.6	12.3	14.2	13.8	12.2	13	13.5	15.2	14.4	16.8	15.8
20	23-3	0	0	0	0	0	0	0	0	0	0	0	0
22	23-4	0	0	0	0	0	0	0	0	0	0	0	0
23	235-	0	0	0	0	0	0	0	0	0.2	0	0	0.1
25	24-3	3.1	0.9	0.5	2.2	0.6	0.5	2.4	1.9	1.2	1.1	0.5	1.1
27	26-3	5.2	5.3	6.1	4.8	4.9	5.9	5.7	4.3	7.1	5.3	4.1	7
28	24-4	1.5	0.8	0.8	0.7	0.4	0.4	1.3	1.1	1.3	0.3	0.3	1.2
31	25-4	0.3	0.4	0.3	0.5	0.4	0.3	0.2	0.4	0.3	0.5	0.3	0.4
32	26-4	3.5	5.1	4.9	3.4	5.2	5.1	9.3	24.2	4.1	9.2	23.9	4.1
42	23-24	0.0	0.0	0.2	0.0	0.2	0.0	0.0	0.1	0.0	0.2	0.0	0.1
43	235-2	0.1	0.0	0.2	0.0	0.0	0.0	0.2	0.1	0.2	0.0	0.0	0.1
47	24-24	8.7	8.3	7.7	9.7	9.5	8.9	9.8	15.3	5.7	10.9	15.6	6.9

49	24-25	2.0	1.7	1.0	2.5	1.8	1.5	1.8	2	1.2	2.5	1.8	1.7
50	246-2	0	0	0	0	0	0	0	0	0	0	0	0
51	24-26	4.8	5.4	6.1	5.2	5.9	6.9	5.1	6.3	3.3	5	3.8	3.4
52	25-25	1.6	1.0	0.7	1.5	0.8	0.6	1.1	0.9	0.8	1.1	0.4	0.8
53	25-26	1.6	1.3	1.6	1.4	1.1	1.9	1.5	0.5	1.8	1.5	0.4	1.8
55	234-3	0	0	0	0	0	0	0	0	0	0	0	0
56	23-34	0	0	0	0	0	0	0	0	0	0	0	0
60	234-4	0	0	0	0	0	0	0	0	0	0	0	0
61	2345-	0.1	0.1	0.1	0	0	0.0	0.1	0	0.1	0	0	0
63	235-4	0.4	0.4	0.5	0.2	0.2	0.2	0.5	0.4	0.5	0.1	0.1	0.4
64	236-4	0.3	0.3	0.3	0.2	0.2	0.2	0.3	0.3	0.3	0.2	0.2	0.2
66	24-34	0.2	0.2	0.1	0.1	0.1	0.0	0.2	0.2	0.1	0.1	0	0
71	26-34	0.3	0.3	0.3	0.2	0.2	0.2	0.3	0.3	0.2	0.1	0.1	0.1
74	245-4	0	0	0	0	0	0	0	0	0	0	0	0
83	235-23	0	0	0	0	0	0	0.1	0	0	0	0	0
90	235-24	0.7	0.9	1.3	0.4	0.6	0.9	1.6	0.9	1.2	1.1	0.4	0.9
91	236-24	0.1	0.1	0.1	0.0	0.1	0.0	0.2	0.1	0.1	0.1	0	0
95	236-25	0	0	0	0	0	0	0	0	0	0	0	0
99	245-24	0.1	0.1	0.2	0.1	0.1	0.1	0.2	0.1	0.2	0	0.1	0.1
101	245-25	0.1	0.0	0.1	0.0	0.0	0.1	0.1	0.1	0.1	0.1	0.1	0.1
102	245-26	0.0	0.0	0.0	0.0	0.0	0.0	0	0	0	0	0	0
105	234-34	1.0	1.0	0.9	0.5	0.5	0.5	1.0	1.0	0.7	0.5	0.4	0.4
114	2345-4	0.1	0.2	0.1	0.1	0.1	0.1	0.1	0.1	0.1	0.1	0.1	0.1
128	234-234	0	0	0	0	0	0	0	0	0	0	0	0
129	2345-23	0	0	0	0	0	0	0	0	0	0	0	0
130	234-235	0	0.0	0.1	0	0	0	0.1	0	0.1	0	0	0
137	2345-24	0.1	0.1	0.1	0	0	0.0	0.1	0	0.1	0	0	0
138	234-245	0	0	0	0	0	0	0	0	0	0	0	0
149	236-245	1.0	1.0	1.2	0.4	0.4	0.4	1.6	1.1	1.1	0.5	0.4	0.4
153	245-245	1.2	1.2	1.4	0.5	0.5	0.5	1.6	1.3	1.1	0.6	0.5	0.5
170	2345-234	1.6	1.6	1.9	0.6	0.6	0.7	2.1	1.7	1.8	0.8	0.6	0.7
Total Amount		**151.0**	**151.1**	**151.7**	**145.5**	**144.6**	**148.2**	**149.5**	**150.9**	**149.2**	**146.1**	**148**	**142.9**

Carbon sources, acetate or the fatty acid mixture were amended to triplicate G-1 microcosm bottles (denoted as A, B, C for acetate and D, E, F for the fatty acid mixture) after the sampling at Week 24, then repeatedly amended twice at Week 30 and Week 36 respectively. Unlike other destructive samplings, the same microcosm bottles were incubated and sampled at Week 36 and Week 51 to continuously track the shifts of individual PCB congeners in the same bottle. Seen in **Table S8**, *ortho* dechlorination products PCB 13 and PCB 15 were observed at Week 36 with carbon sources, which was 15 weeks ahead comparing to the sediment microcosms without carbon source addition. In fatty acids amended microcosms, from Week 36 to Week 51, PCB 13 increased from 0.9 to 1.8 nmole/g in bottle D, 0.8 to 1.9 nmole/kg in bottle E and 0.0 to 0.7 nmole/g in bottle F. In the meanwhile, its parent PCB 25 decreased from 2.4 to 1.1 nmole/kg in bottle A, and 1.9 to 0.5 nmole/g in bottle B, but only 1.2 to 1.1 nmole/g in bottle C. PCB 15 increased from 4.2 to 5.3 nmole/g in bottle D, 5.6 to 7.0 nmole/g in bottle E and 0.4 to 1.0 nmole/g in bottle F. While, its parent PCB 28 decreased from 1.3 to 0.3 nmole/kg in bottle D, and 1.1 to 0.3 nmole/g in bottle E, but only 1.3 to 1.2 nmole/g in bottle F. Structurally, PCB 25 (24-3-CB) is the second generation dechlorination product of PCB 105 (234-34-CB) and PCB 28 (24-4-CB) is the second generation dechlorination product of both PCB 105 and PCB 114 (2345-4-CB). Thus, the rough mass balance between PCB 25 and PCB 13, PCB 28 and PCB 15 only work when the reduction of residual parents PCB 105/PCB 114 and first generation dechlorination products PCB 55/PCB 56/PCB 60/PCB 63/PCB 66/PCB 74 were very small. At Week 36, PCB 105 was 1.0, 1.0 and 0.7 nmole/g in bottle D, E and F, while at Week 51, PCB 105 was 0.5, 0.5 and 0.4 nmole/kg. From Week 36 to Week 51, PCB 114 remained at about 0.1 nmole/g. Other first generation congeners were either below 0.1 nmole/g or reduced by less than 0.3 nmole/g from Week 36 to Week 51 (**Table S8**). Therefore, the *ortho* dechlorination pathways from PCB 25 to PCB 13 and PCB 28 to PCB 15 were definitely present. However, the *ortho* dechlorination activity in bottle F were relatively limited than that of bottle D and bottle E. Looking into the congener concentrations at Week 24, PCB 1 (2-CB) was much higher in bottle F (39.0 nmole/g) than that in bottle D (27.7 nmole/g) and

bottle E (26.2 nmole/g). However, the spiked parent PCB 5 (23-CB) was only 25.9 nmole/g, which indicates that the removal of all *meta*/*para* chlorines on PCB 105/PCB 114 had extensively occurred in bottle F. Thus, the remaining substrate (PCB 25 and PCB 28) available for *ortho* dechlorination was limited after 24 weeks. Moreover, the reduction of PCB 1 from Week 36 to Week 51 was very likely associated with *ortho* dechlorination. Similar accumulation of PCB 13 and PCB 15 and reduction of PCB 1 were also observed in acetate amended bottle A, B and C. Generally, the earlier *ortho* dechlorination activity indicates microorganisms responsible for *ortho* dechlorination may utilize acetate and/or propionate, butyrate as their preferred carbon source and/or energy source.

Table S9. PCB congener concentrations in acetate and the fatty acid mixture amended G-1-Fe at Week 36 and 51 (nmole/g).

IUPAC#	Structure	G-1 Fe AC Week 36			G-1-Fe AC Week 51			G-1-Fe CS Week 36			G-1-Fe CS Week 51		
		Fe-A	Fe-B	Fe-C	Fe-A	Fe-B	Fe-C	Fe-D	Fe-E	Fe-F	Fe-D	Fe-E	Fe-F
1	2-	23.8	23.5	23.9	21.7	21.1	22.9	23.6	23.7	24.3	22.7	20.4	23.6
2	3-	0	0	0	0	0	0	0	0	0	0	0	0
3	4-	11.1	10.6	10.6	11.1	11.5	9.3	10.7	10.2	10.6	9.5	10.2	8.9
4	2-2	0.0	0.0	0.0	0.0	0.0	0.0	0	0	0	0	0	0.7
5	23-	0.3	0.3	0.3	0.2	0.2	0.3	0.3	0.3	0.4	0.3	0.3	0.2
6	2-3	1.8	3.0	2.5	2.2	3.5	2.9	1.0	2.3	2.6	1.4	2.5	2.8
7	24-	0.2	0.1	0.1	0.0	0.0	0.1	0.1	0.2	0.1	0	0	0
8	2-4	1.0	0.7	0.8	1.3	1.0	0.9	1.6	0.9	0.8	1.8	1.0	1.0
9	25-	0	0	0	0	0	0	0	0	0	0	0	0
10	26-	0	0	0	0	0	0	0	0	0	0	0	0.2
12	34-	0.4	0.4	0.4	0.3	0.4	0.4	0.5	0.4	0.5	0.3	0.4	0.3
13	3-4	0.1	0.6	3.8	13.7	15.9	15.5	0.6	1.3	0.6	6.9	15.0	16.1
15	4-4	0.5	2.0	3.4	8.3	5.9	7.0	1.5	1.6	1.7	15.9	7.9	6.7
16	23-2	0	0	0	0	0	0	0	0	0	0	0	0
17	24-2	0.2	0.3	0.2	0.3	0.4	0.4	0.2	0.3	0.2	0.3	0.6	0.9
18	25-2	0.4	1.0	0.8	0.6	1.5	1.2	0.3	0.7	0.8	0.4	1.2	1.4
19	26-2	0	0	0	0	0	0	0	0.2	0.1	0.2	1.3	4.8
20	23-3	0	0	0	0	0	0	0.1	0	0	0	0	0
22	23-4	0	0	0	0	0	0	0	0	0	0	0	0
23	235-	0	0	0	0	0	0	0	0	0	0	0	0
25	24-3	16.5	17.0	13.5	3.0	1.7	1.0	7.3	15.4	17.0	1.6	1.4	1.2
27	26-3	0.4	1.8	2.6	0.5	2.0	2.4	1.1	1.7	3.3	1.0	1.8	3.2
28	24-4	7.8	4.2	3.3	0.5	0.2	0.3	15.7	6.4	4.7	0.3	0.2	0.2
31	25-4	0.1	0.5	0.6	0.8	1.3	1.3	0.2	0.2	0.2	0.4	0.9	0.9
32	26-4	30.4	29.1	28.4	30.6	29.4	28.9	29.9	29.3	27.9	30.2	29.5	28.1
42	23-24	0.2	0.1	0.2	0	0	0	0.2	0.1	0.2	0	0	0
43	235-2	0.0	0.2	0.1	0	0	0	0	0.2	0.2	0	0	0
47	24-24	18.9	11.8	12.5	20.7	12.6	13.3	22.7	13.6	11.5	24.5	14.1	12.3

49	24-25	5.9	7.1	7.1	6.6	8.6	8.9	3.2	7.1	7.4	4.1	8.3	8.6
50	246-2	0	0	0.2	0	0.3	0.2	0.1	0.2	0.2	0.0	0.2	0.0
51	24-26	19.3	19.4	19.5	20.2	20.3	20.1	19.5	18.9	18.3	19.8	18.8	15.0
52	25-25	2.7	8.0	7.3	2.9	8.1	7.1	1.7	6.6	7.7	1.6	6.3	6.9
53	25-26	0.7	0.6	0.6	0.4	0.4	0.6	0.4	0.8	1.2	1.1	0.7	1.0
55	234-3	0	0	0	0	0	0	0	0	0	0	0	0
56	23-34	0	0	0	0	0	0	0	0	0	0	0	0
60	234-4	0	0	0	0	0	0	0	0	0	0	0	0
61	2345-	0	0	0	0	0	0	0	0	0	0	0	0
63	235-4	0.7	0.9	0.7	0.2	0.1	0.1	0.9	0.8	0.7	0.1	0.1	0.1
64	236-4	0.4	0.4	0.4	0.2	0.2	0.2	0.4	0.3	0.4	0.2	0.2	0.2
66	24-34	0.2	0.1	0.1	0.0	0.0	0.0	0.2	0.1	0.2	0.1	0.0	0.0
71	26-34	0.6	0.7	0.5	0.2	0.2	0.2	0.5	0.7	0.6	0.2	0.2	0.2
74	245-4	0	0	0	0	0	0	0	0	0	0	0	0
83	235-23	0	0	0	0	0	0	0	0	0.1	0	0	0
90	235-24	1.3	1.8	2.1	0.6	0.7	0.8	1.6	2.1	2.2	0.7	0.9	0.8
91	236-24	0.1	0.1	0.1	0	0	0	0.1	0.1	0.1	0.1	0	0
95	236-25	0	0	0	0	0	0	0.0	0.0	0.0	0.0	0	0
99	245-24	0.2	0.1	0.1	0	0	0	0.2	0.1	0.2	0.1	0.0	0.1
101	245-25	0.1	0.1	0.2	0	0	0	0.2	0.2	0.1	0	0	0
102	245-26	0.2	0.2	0.2	0	0	0	0.2	0.2	0.1	0	0	0
105	234-34	0.9	0.9	0.8	0.4	0.4	0.4	0.9	0.7	0.8	0.4	0.4	0.4
114	2345-4	0.1	0.1	0.1	0.1	0.0	0.1	0.1	0.1	0.1	0.1	0.1	0.1
128	234-234	0	0	0	0	0	0	0	0	0	0	0	0
129	2345-23	0	0	0	0	0	0	0	0	0	0	0	0
130	234-235	0.1	0.1	0.0	0	0	0	0.0	0.0	0.1	0	0	0.0
137	2345-24	0.1	0.0	0.1	0	0.0	0	0.1	0.0	0.1	0	0.0	0.0
138	234-245	0	0	0.0	0	0	0	0.0	0	0	0	0	0
149	236-245	0.9	1.1	1.2	0.5	0.4	0.5	1.0	1.2	1.3	0.4	0.5	0.5
153	245-245	1.2	1.1	1.0	0.5	0.5	0.4	1.1	1.0	1.1	0.5	0.7	0.5
170	2345-234	1.6	1.3	1.3	0.6	0.6	0.6	1.5	1.3	1.4	0.6	0.6	0.6
Total Amount		**151.6**	**151.4**	**151.4**	**149.3**	**149.3**	**148.1**	**151.4**	**151.6**	**151.7**	**147.7**	**146.7**	**148.3**

In the triplicate acetate amended G-1-Fe microcosms, PCB 13 concentrations increased from 0.1, 0.6 and 3.8 nmole/g slurry at Week 36 to 13.7, 15.9 and 15.5 nmole/g slurry, respectively at Week 51. PCB 15 concentrations increased from 0.5, 2.0 and 3.4 nmole/g slurry at Week 36 to correspondingly 8.3, 5.9 and 7.0nmole/g slurry at Week 51. Meanwhile, PCB 25 concentrations decreased from 16.5, 17.0 and 13.5 nmole/g slurry at Week 36 to correspondingly 3.0, 1.7 and 1.0 nmole/g slurry at Week 51. PCB 28 concentrations decreased from 7.8, 4.2 and 3.3 nmole/g slurry at Week 36 to correspondingly 0.5, 0.2 and 0.3 nmole/g slurry at Week 51. In the triplicate fatty acid mixture amended G-1-Fe microcosms, PCB 13 concentrations increased from 0.6, 1.3 and 0.6 nmole/g slurry at Week 36 to correspondingly 6.9, 15.0 and 16.1 nmole/g slurry at Week 51. PCB 15 concentrations increased from 1.5, 1.6 and 1.7 nmole/g slurry at Week 36 to correspondingly 15.9, 7.9 and 6.7 nmole/g slurry at Week 51. Also, PCB 25 concentrations decreased from 7.3, 15.4 and 17.0 nmole/g slurry at Week 36 to correspondingly 1.6, 1.4 and 1.2 nmole/g slurry at Week 51. PCB 28 concentrations decreased from 15.7, 6.4 and 4.7 nmole/g slurry at Week 36 to correspondingly 0.3, 0.2 and 0.2 nmole/g slurry at Week 51.
